# The Value of Expanding the Training Population to Improve Genomic Selection Models in Tetraploid Potato

**DOI:** 10.3389/fpls.2018.01118

**Published:** 2018-08-06

**Authors:** Elsa Sverrisdóttir, Ea Høegh Riis Sundmark, Heidi Øllegaard Johnsen, Hanne Grethe Kirk, Torben Asp, Luc Janss, Glenn Bryan, Kåre Lehmann Nielsen

**Affiliations:** ^1^Department of Chemistry and Bioscience, Aalborg University, Aalborg, Denmark; ^2^Danespo, Vandel, Denmark; ^3^Department of Molecular Biology and Genetics–Crop Genetics and Biotechnology, Aarhus University, Slagelse, Denmark; ^4^Department of Molecular Biology and Genetics, Center for Quantitative Genetics and Genomics, Aarhus University, Tjele, Denmark; ^5^Cell and Molecular Sciences, The James Hutton Institute, Dundee, United Kingdom

**Keywords:** genomic selection, genomic prediction, *Solanum tuberosum*, potato breeding, dry matter, chipping quality

## Abstract

Genomic selection (GS) is becoming increasingly applicable to crops as the genotyping costs continue to decrease, which makes it an attractive alternative to traditional selective breeding based on observed phenotypes. With genome-wide molecular markers, selection based on predictions from genotypes can be made in the absence of direct phenotyping. The reliability of predictions depends strongly on the number of individuals used for training the predictive algorithms, particularly in a highly genetically diverse organism such as potatoes; however, the relationship between the individuals also has an enormous impact on prediction accuracy. Here we have studied genomic prediction in three different panels of potato cultivars, varying in size, design, and phenotypic profile. We have developed genomic prediction models for two important agronomic traits of potato, dry matter content and chipping quality. We used genotyping-by-sequencing to genotype 1,146 individuals and generated genomic prediction models from 167,637 markers to calculate genomic estimated breeding values with genomic best linear unbiased prediction. Cross-validated prediction correlations of 0.75–0.83 and 0.39–0.79 were obtained for dry matter content and chipping quality, respectively, when combining the three populations. These prediction accuracies were similar to those obtained when predicting performance within each panel. In contrast, but not unexpectedly, predictions across populations were generally lower, 0.37–0.71 and 0.28–0.48 for dry matter content and chipping quality, respectively. These predictions are not limited by the number of markers included, since similar prediction accuracies could be obtained when using merely 7,800 markers (<5%). Our results suggest that predictions across breeding populations in tetraploid potato are presently unreliable, but that individual prediction models within populations can be combined in an additive fashion to obtain high quality prediction models relevant for several breeding populations.

## Introduction

Genomic selection (GS) as a breeding method has become widely used in some livestock industries such as dairy cattle and pig breeding, and it offers new opportunities for increasing the efficiency of plant breeding programs (Meuwissen et al., 2001). It is a form of marker assisted selection (MAS) that predicts breeding values of individuals based on genome-wide molecular markers capturing the small contributions of many loci, whereas traditional MAS uses a limited number of selected markers and thus presumably only captures a small proportion of the total genetic variation. Under GS, using a sufficient number of markers, all genetic variance can potentially be explained, as it is assumed that all quantitative trait loci (QTL) are in linkage disequilibrium with at least one marker (Meuwissen et al., 2001; Goddard and Hayes, 2007). While this assumption is easily met in less genetically diverse organisms such as mammals, it is more challenged in highly diverse organisms such as tetraploid potato. Nonetheless, GS allows for prediction of performance of individuals and subsequent selection of breeding candidates in the absence of direct phenotyping, potentially reducing the breeding cycle, while improving gain from selection and reducing costs associated with phenotyping (Heffner et al., 2009, 2010; Jannink et al., 2010; Slater et al., 2016; Sverrisdóttir et al., 2017).

Today, nearly all new potato varieties emerge from the conventional selective breeding process, where new cultivars are developed from sexual crosses followed by clonal propagation, evaluation, and selection. This process is time-consuming and costly, taking up to 10–15 years from an initial cross to a new variety being released (Halterman et al., 2016). Most potato cultivars are autotetraploid and extremely diverse (Potato Genome Sequencing Consortium et al., 2011; Tomato Genome Consortium et al., 2012). In addition, potato cultivars are highly heterozygous, making prediction of the outcome of a cross particularly difficult (Potato Genome Sequencing Consortium et al., 2011). Yet, potato is the third most important food crop worldwide, and it is the most efficient producer of food energy and nutrition per unit area with similar or less input of nutrients and water compared to cereals (FAOSTAT, 2015). Accelerating the breeding gain of potato is therefore of great interest, not only to potato breeders, but to contribute to future global food security.

In a previous study (Sverrisdóttir et al., 2017), we described the results of genomic prediction of tetraploid potato for starch content and chipping (crisping) quality. Our prediction models included potato plants grown and phenotyped at the same breeding station in Vandel, Denmark. Due to the extremely high allelic diversity of tetraploid potato, genomic prediction models likely necessitate large training populations to efficiently capture all the genetic diversity of elite potato germplasm and enable accurate prediction across the entire spectrum of elite potatoes. On the other hand, if (important) genetic diversity is represented widely across potato breeding material, given the tetraploid nature of potato, more alleles are analyzed per genotype than in diploids, which would decrease the number of individuals needed to be genotyped to obtain a reliable model. The latter argument, however, relies on the assumption that potatoes are generally outbred and that underlying population structure is not causing allele frequencies to be substantially different between breeding populations. Whether this holds true is unknown. Furthermore, it is difficult to envision that a very large training population will be established in a single study, more likely “local” prediction models encompassing regionally, commercially, and historically aggregated material will be developed independently. Therefore in this study, we have investigated whether GS models for potato can be expanded in an additive fashion from models made from smaller distinct training populations to achieve overall good predictive performance, without compromising the predictive performance within each population. To this end, we have genotyped three populations using genotyping-by-sequencing (GBS) and generated genomic prediction models for dry matter content and chipping quality with genomic best linear unbiased prediction (GBLUP). The populations varied in size, phenotypic profile, and environments, where two populations were from Denmark while the third population was from the United Kingdom. We describe the results of genomic prediction within and across the different populations. We also examine the effect of expanding the training population by combining all three populations for prediction models, and finally, we study the effect of marker density on predictions.

## Materials and methods

### Plant material

Seven Hundred and Sixty Two clones were randomly chosen from a mapping population established at the breeding station LKF Vandel in Vandel, Denmark, called the MASPOT population. The MASPOT population consists of roughly 5,000 offspring that were generated by systematic cross-pollination of 18 distinct potato cultivars in a full-diallel crossing design, the parents being either established varieties or advanced breeding clones (see Supplementary File 1 for a detailed description). The selected subset of 762 offspring is referred to as the MASPOT population in this paper. The offspring were planted in the field at Vandel, Denmark in 2013 and 2014 (Sverrisdóttir et al., 2017). Plant density was approximately 40,000 plants/hectare with 30 cm between plants and 75 cm between rows. In 2013, seedling tubers were planted April 24th to 25th (no replicates) in blocks of 24 parcels per block. They were harvested August 12th to 30th (109–128 days after planting). The plants were desiccated 1–2 weeks before harvesting. No checks were used, as tubers from seedling plants cause delayed emerging plant in comparison to seed potatoes. In 2014, a randomized block design was used with two replicates. The offspring were divided into four groups based on earliness of parents. The groups were planted April 24th, 25th, 28th, or 29th, in blocks with 28 parcels per block. They were harvested August 11th to 29th (109–129 days after planting), also with 1–2 weeks of desiccation, where group 1 was harvested first and group 4 was harvested last. As the population was highly diverse, not all plants were fully mature at harvest. Nineteen checks were used, 18 of which were the parents used to generate the population, and they were also planted in two replicates. The checks were observed manually for signs of unusual development/disease infection. No abnormalities were observed and this was taken as indication of credible plant material for all clones. The soil type was Sandy Loam. Fertilization was done with 1,000 kg NPK 14-3-15 per hectare. Pests and diseases were controlled with Fenix and Titus before and right after sprouting (weed), Mospilan in the end of June and again in the end of July (insects), and alternating Ranman and Revus from approximately June 23th and until desiccation as needed, depending on weather (late blight). The fields were irrigated as needed.

Two test panels were used to evaluate the robustness of prediction models and compare performance across different populations. A test panel from Denmark, referred to as Test panel DK, consisted of 92 individuals (see Supplementary File 2) selected from a mixture of elite cultivars and breeding clones that have been grown, harvested and phenotyped in the years 1997–2014 in Vandel, Denmark. Among the chosen cultivars were also the 18 parents to the MASPOT population. The cultivars were planted around mid-April to mid-May and harvested in late August and September, where cultivars used for starch production were harvested last. Tubers were desiccated 1–2 weeks before harvesting and plants were generally fully mature at harvest. Otherwise growing conditions were the same as for the MASPOT panel. Varying amounts of data were available (some years missing) for each cultivar in Test panel DK.

The test panel from UK, referred to hereafter as Test panel UK, consisted of 292 breeding clones and cultivars grown, harvested and phenotyped at two different sites in UK (Cambridge and York) in 2012 and 2013. At each site replicated trials (two replicates) with two nitrogen levels (100 and 200 kg/ha) were conducted according to an alpha design, thereby leading to a total of eight “environments.” Tubers were harvested in September/October >140 days after planting. Overall, twenty quantitative traits were measured, but not all traits were phenotyped in every environment.

The relationship between the three populations is visualized in Supplementary Figure S1.

### Phenotyping and adjustment for environmental effects

Dry matter content for the MASPOT population was determined for individuals harvested in 2013 and 2014 with two replicates in the last year, while dry matter content for Test panel DK was determined for individuals harvested in the years between 1997 and 2014 as described in Sverrisdóttir et al. (2017). The tubers were washed and a basket of 1.5–10 kg of tubers was weighed above and under water, shortly after harvesting. The dry matter content was calculated using the following empirical equation:

$$\begin{array}{l} DM\%=214\cdot \left(\left(\frac{weight\;in\;air}{\left(weight\;in\;air\right)-\left(weight\;in\;water\right)}\right)-0.988\right) \end{array}$$

Dry matter content for Test panel UK was determined for individuals harvested in 2012 and 2013 with two repetitions and was measured similarly to the Danish populations. Tubers were gently washed and air-dried, after which they were placed in a basket and weighed above and under water with a Weltech PW-2050 weighing system (Weltech International Ltd). The percentage dry matter was automatically calculated by the weighing system.

Chipping quality was determined as chip color following frying in oil after cold storage of tubers, although different color scales were used for the Danish populations and Test panel UK. For the MASPOT population, chipping quality was determined for offspring harvested in 2013, while chipping quality for Test panel DK was determined for individuals harvested in the years 1997-2014. Tubers were stored at 4°C (MASPOT population) or 6°C (Test panel DK) for approximately 2 months, after which they were stored at room temperature for 2–6 h prior to frying. Four to six slices (1–2 mm) of each tuber were fried in sunflower oil at 180°C until no more bubbles emerged (typically 2–3 min). Frying color was assessed visually to a standard set on an arbitrary grading scale from 1 (dark) to 9 (light). Chipping quality for Test panel UK was determined for individuals harvested in 2012 and 2013. Tubers were stored at 6°C for three months. Five tubers from each sample were washed and one chip (10 mm width) from each tuber was patted dry and fried in 190°C hot oil for 4 min. The chips were drained and placed on absorbent paper after which they were scored visually on a 1–6 (light-dark) scale. The chipping quality data for Test panel UK was then converted into the 1–9 scale used for the Danish populations, assuming a negative linear correlation between the two scales.

All phenotypic data were corrected for variation across years and location by fitting a linear mixed-effects model to the phenotypic data via restricted maximum likelihood (REML) using the following model:

$$\begin{array}{l} y_{ijk}=\mu +genotype_{i}+year_{j}+location_{k}+e_{ijk} \end{array}$$

where *y*_*ijk*_ is the observed phenotype, μ is the overall mean, *genotype*_*i*_ is the random effect of the *i*th genotype, *year*_*j*_ is the fixed effect of the *j*th year, *location*_*k*_ is the fixed effect of the *k*th location, and *e*_*ijk*_ is the error term. The model was made with the lme4 package in R (Bates et al., 2014; R Core Team, 2015). All three populations were analyzed together in a single analysis and the calculated effects were subtracted from each data point. The mean of corrected data was then found for each genotype. Note that no term for genotype-by-environment (GxE) interactions is included in the model. While there is likely to be relevant GxE interactions, our experimental design does not allow for a rigorous estimation of this. The 2 years of data we have for the main population, MASPOT, are not immediately comparable, since data from the first year is for seedling tubers that cannot readily be compared with data for subsequent tubers without introducing additional error. Furthermore, we have no data at different locations for the MASPOT population. As a consequence, and to decrease the risk of overfitting the data, we have chosen to simplify the model.

### Preparation of genotyping-by-sequencing libraries

GBS libraries were prepared as described in Sverrisdóttir et al. (2017), a protocol adapted from Elshire et al. (2011). Briefly, 5′ and 3′ adapters for Illumina sequencing were designed (see Supplementary File 3), enabling a 96 multiplexing system. DNA extracted from leaf tissue was digested with *Ape*KI and fragments were ligated to adapters, pooled in 96-plex libraries, purified and amplified with PCR. MASPOT libraries were sequenced on a HiSeq 2000 (Illumina, San Diego, USA) with single-read sequencing (100 bp) and each 96-plex library was sequenced on three channels of a flow cell. The Test panel DK library was sequenced on two rapid run flow cells on a HiSeq 2500 (Illumina, San Diego, USA) with single-read sequencing (100 bp). Test panel UK libraries were sequenced on HiSeq 2500 (Illumina, San Diego, USA) on two channels each with single-read sequencing (200 bp).

### Filtering raw sequence data, mapping and SNP calling

Sequenced reads were processed as described in Sverrisdóttir et al. (2017). Reads were demultiplexed, trimmed, and mapped to the potato reference genome sequence (DM v4.03; Sharma et al., 2013). Single nucleotide polymorphisms (SNPs) were called using the Genome Analysis Toolkits (McKenna et al., 2010) UnifiedGenotyper tool and filtered. Rather than calling genotypes, which would require high coverage sequence reads, the variant allele frequencies at each data point were estimated and used directly in further analysis according to Ashraf et al. (2014). Minor allele frequency was estimated from the read coverage, and SNPs were filtered on a minimum minor allele frequency of 1% (average variant allele frequency <0.99 and >0.01). Additionally, SNPs were filtered with a read coverage between 5 and 60 and a maximum number of missing data of 50%. Individuals with greater than 70% missing data were removed. All statistical analysis and graphics were performed in R (R Core Team, 2016).

### Reduced marker sets

Two reduced sets of markers were generated from the filtered marker set. The first set was generated using stringent filtering with a maximum allowed number of missing data of 1% and where all individuals with more than 10% of missing markers were removed. The second reduced data set was generated by selecting 7,800 markers randomly. The random sampling was repeated 10 times.

### Subsets

Subsets were sampled randomly from the MASPOT population and Test panel UK containing either 39 or 80 samples. Each subsampling was repeated 10 times.

### Assessment of genetic diversity across populations

A principal component analysis (PCA) was performed on the genomic relationship matrix to assess population structure using the prcomp tool, a part of the stats package in R (R Core Team, 2016). The genomic relationship matrix (**G**) was created from the genotype matrix (**Z**) according to VanRaden (2008) with **Z** containing allele frequencies for each sample and SNP computed from sequence data (Ashraf et al., 2016). The allele frequencies were values between 0 and 1 calculated as the ratio between allele counts of the alternative allele and the total allele count, hence tetraploid allele dosage will also be captured:

$$\begin{array}{l} AF=\frac{AC_{alt}}{AC_{ref}+AC_{alt}} \end{array}$$

The allele frequencies were corrected for missing data using the following correction as described by VanRaden (2008):

$$\begin{array}{l} w_{i}=\sqrt{\frac{\sum p_{k}\left(1-p_{k}\right)\;over\;all\;loci}{\sum p_{k}\left(1-p_{k}\right)\;over\;only\;non\text{-}missing\;loci}} \end{array}$$

where *p*_*k*_ is the mean allele frequency at locus *k*. The genotype matrix was centered and adjusted for missing values as described by Ashraf et al. (2016), after which missing values were set to zero, corresponding to a mean imputation for missing data.

$$\begin{array}{l} Z_{ik}=\left(X_{ik}-p_{k}\right){\cdot w}_{i} \end{array}$$

where ***X***_*ik*_ is the allele frequency in family *i* at locus *k*. The genomic relationship matrix was scaled using global scaling (VanRaden method 1) (VanRaden, 2008) with a modification to adjust for tetraploids (Ashraf et al., 2014, 2016).

$$\begin{array}{l} \text{G}=\frac{{\text{Z}}^{\prime }\text{Z}}{0.25\sum p_{k}\left(1-p_{k}\right)} \end{array}$$

where 0.25∑*p*_*k*_ (1 − *p*_*k*_) is the sum of genotype variance and also the average diagonal of **Z**'**Z**.

### Genomic prediction models

Genomic estimated breeding values (GEBVs) were calculated with GBLUP. The GBLUP method is the most common used parametric method for GS. It is equivalent with a ridge-regression model with uniform shrinkage of SNP effects regardless of the marker effect size, although shrinkage is dependent on sample size and allele frequency (Gianola, 2013). GBLUP uses the genomic relationship matrix and directly estimates genomic breeding values with the model (Meuwissen et al., 2001).

$$\begin{array}{l} y=1\mu +g+e \end{array}$$

where *y* is a vector of phenotypes, μ is the mean, *e* is a vector of random normal deviates, and ***g*** is a vector of random genomic breeding values with the distribution:

$$\begin{array}{l} g\sim N\left(0,\text{G}\sigma_{g}^{2}\right) \end{array}$$

All models were performed with the BGLR package in R (de los Campos and Perez Rodriguez, 2015) with default settings for priors. 12,000 iterations were used and a burn-in setting of 2000. Predictions were made for each population using one of the other populations for the modeling. In addition, predictions were made within-population for each population, as well as with a combined population, combining all three populations for modeling. All within-population analyses were performed using 5-fold cross-validation schemes. The data were randomly divided into groups and one group was then used as validation set while the remaining groups were used as training population. The process was repeated, each time with another group as validation set, until predictions had been obtained for all individuals. Each analysis was repeated with 50 different cross-validation groupings and the average GEBV over the 50 samplings was taken. The accuracy of the GEBVs was determined as the Pearson correlation between the GEBVs and the observed phenotypes, described in this paper as prediction correlation:

$$\begin{array}{l} r\left(GEBV:y\right) \end{array}$$

The regression of the observed phenotype values on the predicted values was used as a measure of the bias for the GEBVs, where a slope of the regression of β = 1 denotes no bias, β < 1 implies that high GEBVs overestimate the observed phenotypes while low GEBVs underestimate the observed phenotypes, and vice versa for β > 1 (Luan et al., 2009).

## Results

### Genotypes and phenotypes

Sequencing yielded on average 4 million trimmed and filtered reads per sample for the MASPOT population, 1.5 million trimmed and filtered reads per sample for Test panel DK, and 2 million trimmed and filtered reads per sample for Test panel UK. A total of 4.6 million variant sites were found. After filtering, 167,637 SNPs remained (see Supplementary File 4). Twenty-One samples that contained less than 30% of the selected SNPs were removed, resulting in 755 samples remaining in the MASPOT population, 80 samples in Test panel DK, and 290 samples in Test panel UK.

**Figure 1:**
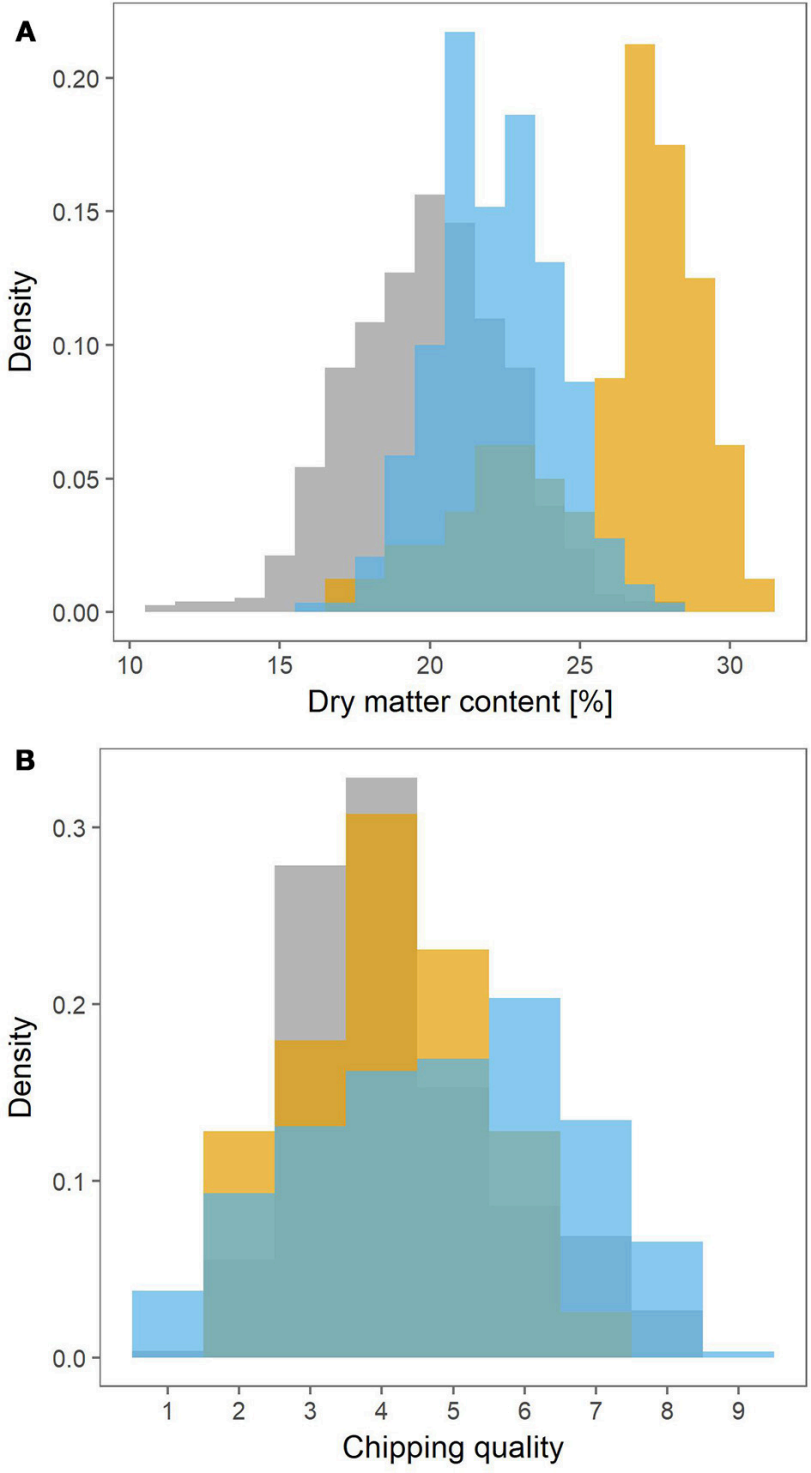
Density histograms depicting the phenotype distributions for the MASPOT population (gray), Test panel DK (yellow), and Test panel UK (blue). Dry matter content **(A)** was measured as percentage, while chipping quality **(B)** was determined as assessment of frying color on a scale from 1 (poor) to 9 (best).

The MASPOT population had the widest range in dry matter content, ranging from 11% to 28% (Figure 1A). The lowest dry matter content in Test panel UK and Test panel DK however were 16 and 17%, respectively, and with maximums at 28 and 30%. Test panel DK clearly had the highest average dry matter content, with a density histogram shifted toward the right, while MASPOT and Test panel UK had more similar histogram profiles with Test panel UK shifted slightly to higher dry matter content.

Since two different color scales were used to evaluate chipping quality for the Danish populations and Test panel UK, the 1–6 (light-dark) scale for Test panel UK was converted into the 1–9 (dark-light) scale used for the MASPOT population and Test panel DK assuming a linear negative correlation between the two scales. The converted values for Test panel UK were between 0.9 and 8.52, while the MASPOT population and Test panel DK had values between 1 to 8 and 2 to 7.5, respectively (Figure 1B).

### Assessment of genetic diversity across populations

**Figure 2:**
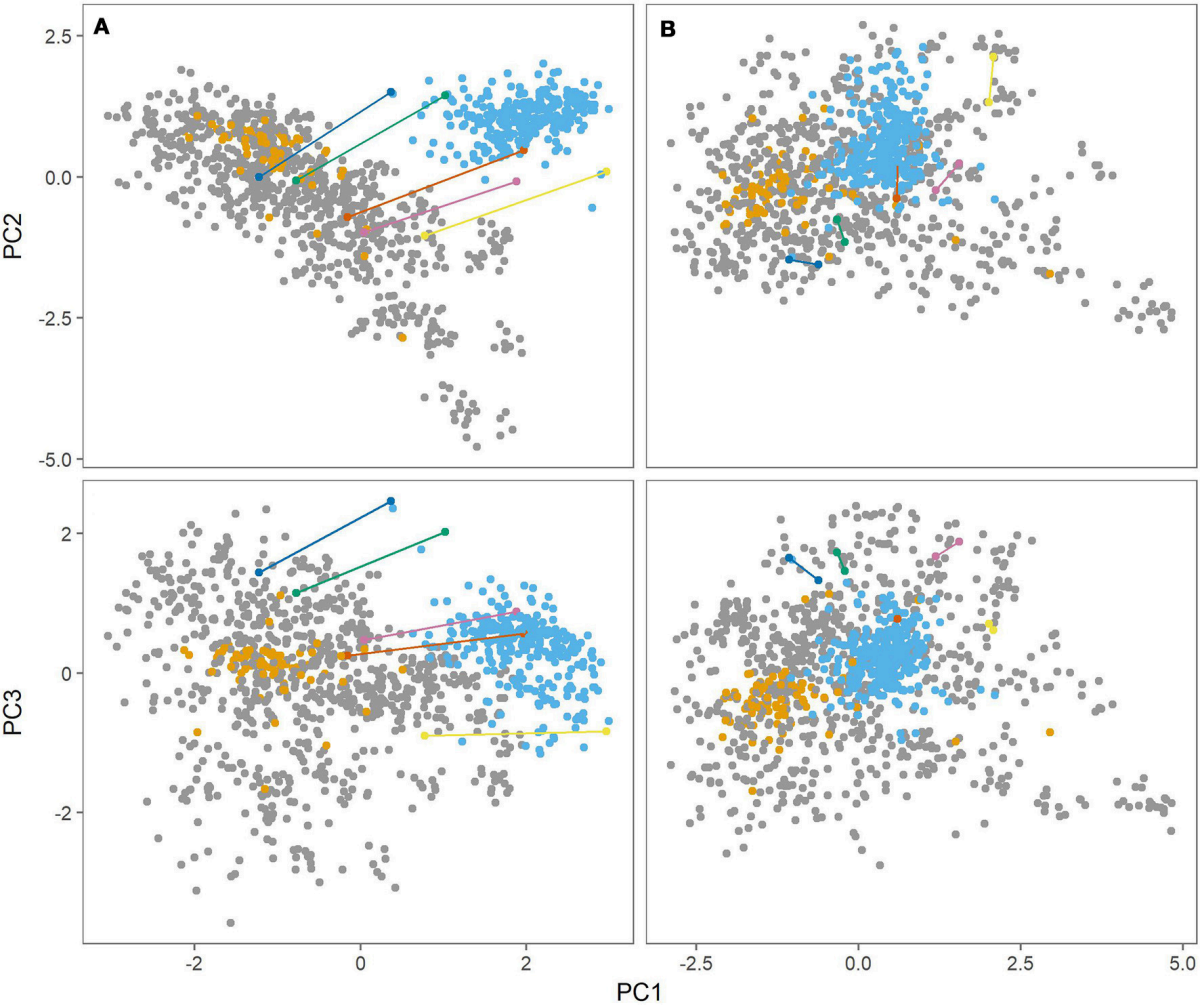
Principal component analysis (PCA) of genomic relationship matrix constructed from genotypes at 167,637 SNP markers **(A)** and 7,800 markers **(B)** for the three populations, MASPOT (gray), Test panel DK (yellow), and Test panel UK (blue). The first principal component (PC1) is plottet against the second principal component (PC2) in the top and against the third principal component (PC3) in the bottom. Plotted in various colors and connected with lines are five individuals that were genotyped in both Test panel DK and Test panel UK. The three components account for 19.8, 13.4, and 8.6% of the explained variance, respectively, in regards to the 167,637 markers, and 19.7, 11.4, and 9.2%, respectively, for the 7,800 marker set.

Figure 2 (left panel) shows the first three principal components from the PCA. There was a clear distinction between the Danish populations (MASPOT and Test panel DK) and Test panel UK. There was also a clear grouping of Test panel DK, overlapping with the MASPOT population. Since Test panel DK contained the parents that were used to generate the MASPOT population and was of “Danish” decent, this was expected. While some difference between the Danish populations and Test panel UK could be expected, it was curious and unexpected that there was such a significant difference between the genotypes. Test panel DK and Test panel UK had five potato varieties in common, and these five varieties were therefore genotyped as a part of both the Test panel DK as well as Test panel UK. Bearing in mind that the PCA plot only contains genotypic data, it should be expected that the genotype for each of the five individuals would be the same, regardless of whether it was genotyped as part of Test panel DK or Test panel UK; however, in the PCA plot, two distinct points were present for each individual, one for each population. In fact, it seemed that there was a parallel dislocation in the PCA plot between Test panel UK and the rest of individuals. The same variation was observed in PCA plots with balanced data, where subsets of 80 individuals were sampled from each population (see Supplementary Figures S2–S11). This suggested that the observed diversity, at least in part, stemmed from experimental variation during sample preparation, sequencing and/or data analysis. In GBS, the genotyping sites generated from the restriction enzymes are undersampled and therefore two samples are likely to be different (but both representative) between two repetitions or separate preparations. Prompted by this line of thought, in a parallel experiment we stringently filtered SNPs, removing all markers with >1% missing data, and removing all samples with >10% missing markers, reducing the marker number from 167,637 to 7,800. Following PCA (Figure 2, right panel) the populations merged into the diversity defined by the MASPOT population, but there was still a clear grouping of both Test panel UK and Test panel DK within this diversity. No significant loss of variance explained by the first three components was observed: 41.8 and 40.2, respectively. Though the five individuals common to both populations did not have exactly the same location in the PCA plot, indicating that there was still some variation in the estimation of genetic variation as expected from the undersampling nature of GBS, they were located much closer together and no longer showed a parallel shift in the PCA plots. In either case, the largest genetic variation observed was within the MASPOT population, in regard to all three PCs.

### Genomic prediction models

GEBVs were calculated for each individual with GBLUP (VanRaden, 2008) using all 167,637 markers. For each population, GEBVs were calculated within-population, using a 5-fold cross-validation system. To test robustness of the Bayesian methods used for the cross-validation, multiple runs of the same cross-validation fold was performed, and only insignificant variation was observed confirming robustness of the cross validation method (data not shown). A leave-one-out cross-validation scheme was also applied with similar results (see Supplementary Table S1). In addition, to evaluate the prediction performance across populations, GEBVs were calculated for each population, using one of the other populations for modeling. Finally, a model was made where all three populations were combined and used as training population and GEBVs were calculated for each individual with a 5-fold cross-validation system. In the following, results from these calculations using all populations combined will be referred to as “combined population.” Each prediction was repeated 50 times or with 50 different cross-validation groupings.

In general, higher prediction correlations were observed for dry matter (0.75–0.83, combined population) than for chipping quality (0.49–0.78, combined population) (Table 1). Best prediction correlations for MASPOT and Test panel UK were obtained within-population or using the combined population. For chipping quality, somewhat higher prediction correlations were found for Test panel UK than for the MASPOT population (0.78–0.79 and 0.56–0.57, respectively), while the prediction correlations were similar for both populations with regards to dry matter content (0.72–0.75 and 0.74–0.75, respectively). In both cases, the predictions were similar when using the same population as training and test population or using the combined population. In contrast, Test panel DK had lower prediction correlations when using Test panel DK as training population compared to the combined population for chipping quality. This was likely caused by the lower number of individuals with chipping quality data available in the Test panel DK (*n* = 39) compared to the other training populations (MASPOT *n* = 524 and Test Panel UK *n* = 290, respectively).

**Table 1:** Mean prediction correlations and bias found with GBLUP over 50 repeats with 167,637 markers, using the three populations separately and combined for modeling.

**Prediction set/ training set**	**MASPOT**	**Test panel DK**	**Test panel UK**	**Combined**
**DRY MATTER**				
MASPOT [755]	**0.74 [1.04]**	0.67 [1.41]	0.62 [1.55]	0.75 [0.99]
Test panel DK [80]	0.71 [1.91]	**0.82 [1.46]**	0.63 [2.85]	0.83 [1.08]
Test panel UK [290]	0.57 [1.64]	0.37 [2.20]	**0.72 [1.54]**	0.75 [1.36]
**CHIPPING QUALITY**				
MASPOT [524]	**0.56 [1.12]**	0.35 [1.31]	0.30 [0.31]	0.57 [0.99]
Test panel DK [39]	0.48 [1.76]	**0.27 [1.74]**	0.42 [0.63]	0.49 [1.30]
Test panel UK [290]	0.43 [2.04]	0.28 [3.79]	**0.79 [1.54]**	0.78 [1.47]

*The population used for training the model is listed horizontally while the predicted population is listed vertically. Bias is listed in brackets. The number of phenotypes available in each case is indicated with brackets. Bold lettering indicates within-population predictions, where the same population was used for training and test population with 5-fold cross-validation*.

**Figure 3:**
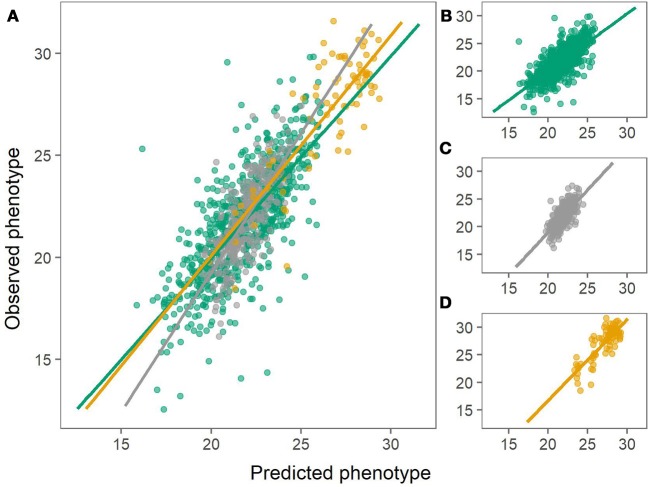
Predictions of dry matter content using the combined population (left) or using within-population predictions (right). Green: Predictions of MASPOT population. Gray: Predictions of Test panel UK. Yellow: Predictions of Test panel DK. **(A)** Combined population. **(B)** MASPOT model. **(C)** Test panel UK model. **(D)** Test panel DK model.

**Figure 4:**
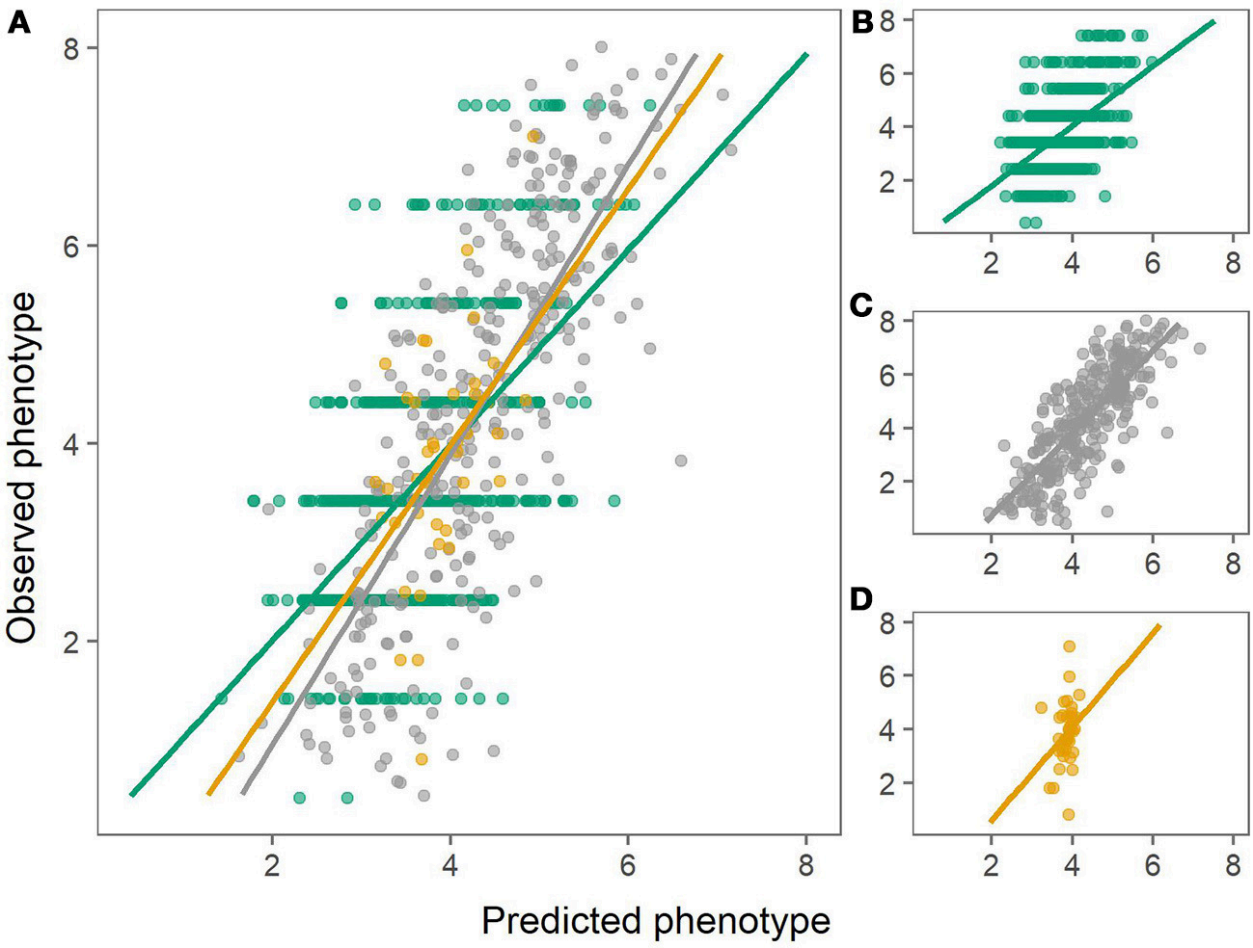
Predictions of chipping quality using the combined population (left) or using within-population predictions (right). Green: Predictions of MASPOT population. Gray: Predictions of Test panel UK. Yellow: Predictions of Test panel DK. **(A)** Combined population. **(B)** MASPOT model. **(C)** Test panel UK model. **(D)** Test panel DK model.

The prediction biases varied for each population, evaluated from the slope (β) of the regression line between the observed (*y*) and the predicted values (*x*), where β of 1 indicates no bias. In general, biases for the combined population were larger (greater absolute deviation from 1) than for within-population predictions for both chipping quality and dry matter (Figures 3, 4, Table 1).

The prediction ability across populations (using one population as training set and another as test population) was generally lower than when using the same population as training and test population or the combined population. In most cases, especially for chipping quality predictions, the observed bias for the GEBVs estimated across populations was rather large, indicating a deflation (β > 1) or inflation (β < 1) of predicted values relative to the observed phenotypes.

### Prediction correlation using a marker set of 7,800 markers

As was clear from the PCA, there was a difference in the set of markers used to predict the Danish populations and Test panel UK when using all 167,637 markers that was diminished when using a more stringent filtering. The prediction models were therefore repeated with a “cherry-picked” marker set of 7,800, where only markers with <1% missing data were used, and where all samples contained at least 90% of the selected markers (see Supplementary File 5). Somewhat surprisingly, only small or negligible differences were to be found for most prediction correlations (Table 2, Supplementary Figure S12, S13), and in some cases, the predictions with the smaller marker set even led to smaller observed bias. Models using the combined population generally gave slightly lower prediction correlations and also lower slopes, but slightly larger bias, except for the UK panel, where the bias was smaller using the smaller number of markers. Nonetheless, both marker sets gave comparable, good prediction accuracies.

**Table 2:** Mean prediction correlations and bias found with GBLUP over 50 repeats with 7,800 cherry-picked markers, using the three populations separately and combined for modeling.

**Prediction set/ training set**	**MASPOT**	**Test panel DK**	**Test panel UK**	**Combined**
**DRY MATTER**				
MASPOT [755]	**0.73 [1.01]**	0.67 [0.87]	0.59 [1.16]	0.74 [0.93]
Test panel DK [90]	0.72 [1.47]	**0.78 [1.34]**	0.66 [1.74]	0.79 [0.99]
Test panel UK [289]	0.46 [1.18]	0.44 [1.13]	**0.72 [1.29]**	0.71 [1.16]
**CHIPPING QUALITY**				
MASPOT [524]	**0.51 [1.03]**	0.37 [0.75]	0.30 [0.21]	0.49 [0.63]
Test panel DK [42]	0.53 [1.09]	**0.26 [1.25]**	0.37 [0.25]	0.39 [0.41]
Test panel UK [289]	0.44 [3.04]	0.28 [3.04]	**0.79 [1.30]**	0.78 [1.39]

*The population used for training the model is listed horizontally while the predicted population is listed vertically. Bias is listed in brackets. The number of phenotypes available in each case is indicated with brackets. Bold lettering indicates within-population predictions, where the same population was used for training and test population with 5-fold cross-validation*.

To test if the selection process of the smaller marker set to ensure high representation in all clones was important to obtain high accuracy, 7,800 random markers were also selected (Supplementary File 6). Intriguingly and surprisingly, the random marker set predicted similarly to the cherry-picked set (Table 3, Supplementary Figures S14, S15). However, the predictions and the bias in particular using the smaller marker sets depended highly on which set of 7,800 markers were selected from the original set, as seen from the variance of the repeated samplings of marker sets in Supplementary Figures S16, S17. The variance was greatest for the two test panels.

**Table 3:** Mean prediction correlations and bias with standard deviations found with GBLUP over 50 repeats with 7,800 randomly selected markers and with 10 different sets of markers, using the three populations separately and combined for modeling.

**Prediction set/training set**	**MASPOT**	**Test panel DK**	**Test panel UK**	**Combined**
**DRY MATTER**				
MASPOT [755]	**0.74** ±**0.01 [1.03** ±**0.00]**	0.65 ± 0.01 [1.34 ± 0.03]	0.57 ± 0.02 [1.38 ± 0.08]	0.74 ± 0.01 [0.95 ± 0.01]
Test panel DK [78–81]	0.70 ± 0.03 [1.86 ± 0.11]	**0.79** ±**0.01 [1.43** ±**0.02]**	0.57 ± 0.06 [2.35 ± 0.27]	0.81 ± 0.01 [1.03 ± 0.02]
Test panel UK [290]	0.49 ± 0.04 [1.30 ± 0.13]	0.32 ± 0.04 [1.53 ± 0.17]	**0.70** ±**0.02 [1.43** ±**0.03]**	0.68 ± 0.02 [1.15 ± 0.04]
**CHIPPING QUALITY**				
MASPOT [524]	**0.52** ±**0.01 [1.05** ±**0.01]**	0.33 ± 0.02 [1.21 ± 0.10]	0.29 ± 0.02 [0.43 ± 0.09]	0.51 ± 0.02 [0.80 ± 0.03]
Test panel DK [38–39]	0.43 ± 0.07 [1.38 ± 0.28]	**0.34** ±**0.04 [1.38** ±**0.15]**	0.36 ± 0.10 [0.73 ± 0.26]	0.49 ± 0.05 [1.01 ± 0.16]
Test panel UK [290]	0.33 ± 0.03 [1.79 ± 0.50]	0.24 ± 0.05 [3.35 ± 1.32]	**0.77** ±**0.02 [1.46** ±**0.03]**	0.76 ± 0.02 [1.45 ± 0.03]

*The population used for training the model is listed horizontally while the predicted population is listed vertically. Bias is listed in brackets. The number of phenotypes available in each case is indicated with brackets. Bold lettering indicates within-population predictions, where the same population was used for training and test population with 5-fold cross-validation*.

### Prediction correlation using subsets

Since the three populations are very different in size, subsets were made containing either 39 or 80 samples, corresponding to the number of samples in the smallest population, Test panel DK (*n* = 80) (39 as the number of samples with chipping quality data available). In general, prediction correlations were lower when using subsampling of the populations, as expected when using (too) small training populations (Table 4, Supplementary Figure S18, S19). In a few cases, prediction correlations were similar or higher compared to when using the whole populations of MASPOT or Test panel UK, but in most cases, biases were larger (Supplementary Figure S20, S21). The largest drop in prediction correlations were seen when predicting chipping quality in Test panel UK and the MASPOT population, especially when predicting within population. No substantial differences were seen when using Test panel DK as training population, however, larger biases were obtained.

**Table 4:** Mean prediction correlations and bias with standard deviations found with GBLUP over 50 repeats with 167,637 markers and with 10 different subsampling of either 39 or 80, using the three populations separately and combined for modeling.

**Prediction set/training set**	**MASPOT**	**Test panel DK**	**Test panel UK**	**Combined**
**DRY MATTER**				
MASPOT [80]	**0.70** ±**0.08 [1.53** ±**0.10]**	0.66 ± 0.05 [1.52 ± 0.14]	0.50 ± 0.08 [2.63 ± 0.45]	0.71 ± 0.07 [1.05 ± 0.06]
Test panel DK [80]	0.67 ± 0.04 [2.78 ± 0.22]	**0.82 [1.46]**	0.53 ± 0.10 [5.21 ± 0.69]	0.82 ± 0.00 [1.11 ± 0.02]
Test panel UK [80]	0.36 ± 0.08 [1.86 ± 0.47]	0.42 ± 0.11 [2.71 ± 0.68]	**0.53** ±**0.07 [2.54** ±**0.34]**	0.60 ± 0.06 [1.73 ± 0.25]
**CHIPPING QUALITY**				
MASPOT [39]	**0.18** ±**0.22 [0.78** ±**1.03]**	0.36 ± 0.13 [1.71 ± 0.55]	0.24 ± 0.20 [1.03 ± 0.87]	0.37 ± 0.13 [1.04 ± 0.32]
Test panel DK [39]	0.34 ± 0.13 [2.23 ± 0.96]	**0.27 [1.74]**	0.40 ± 0.17 [2.52 ± 1.09]	0.52 ± 0.08 [1.82 ± 0.28]
Test panel UK [39]	0.25 ± 0.22 [5.32 ± 4.98]	0.29 ± 0.19 [5.85 ± 4.33]	**0.32** ±**0.19 [2.28** ±**1.49]**	0.44 ± 0.13 [2.77 ± 0.78]

*The population used for training the model is listed horizontally while the predicted population is listed vertically. Bias is listed in brackets. The number of phenotypes available in each case is indicated with brackets. Bold lettering indicates within-population predictions, where the same population was used for training and test population with 5-fold cross-validation. No subsampling was performed in Test panel DK, being the smallest population, hence no standard deviations are presented*.

## Discussion

### Assessment of genetic diversity across populations

Three panels were used in this study: two panels grown and phenotyped in Denmark, and one panel grown and phenotyped in the UK. Variations between years and locations were corrected for with a linear mixed-effects model. This would presumably also correct for differences in measurement methods to some extent, however there may be variations that the model does not take into account. Especially the chipping quality measurements varied between the panels. All panels were phenotyped for chipping quality after cold-induced sweetening, although for the MASPOT population, the harvested tubers were stored at 4°C for approximately two months, while Test panel DK and Test panel UK were stored at 6°C for two to three months or three months, respectively. The difference in storage could have affected the phenotypes, adding to the difficulty of predicting chipping quality across populations. In addition, two different color scales were used to estimate the chipping quality for the Danish populations and Test panel UK, and although it is reasonable to assume a linear correlation between the two scales, the conversion of data from one scale to another likely introduces additional variation in data and may be an important cause for the higher difficulty in obtaining comparable prediction accuracy across and within populations for this trait.

PCA of the genetic markers initially revealed a very distinct grouping of the three populations, and the largest genetic variation was observed within the MASPOT population. The MASPOT population, consisting of crosses generated from 18 tetraploid cultivars and breeding clones, has not been subjected to any selection for performance traits, in contrast to the two test populations, which were commercial breeding populations both consisting of elite cultivars and breeding clones that had been selected for several years for different agronomical important traits. However, the observed genetic diversity is not consistent with the expected underlying genetic diversity. The Test panel DK includes the 18 parents generating MASPOT and thus contains all the genetic diversity of MASPOT as well as 62 additional individuals which adds additional genetic diversity to the Test panel DK. The inflation of the observed genetic diversity is likely caused by the unbalanced sampling of the two populations. Since any sample is an undersampling of the true diversity, each will add new markers to the total set of markers used. In turn, this will add to the diversity observed in the PCA plot. Since almost 10 times more individuals were sampled from MASPOT than for Test Panel DK, the observed genetic variation is larger. Hence the observed diversity from GBS is not immediately a good measurement of the underlying genetic diversity, at least not in unbalanced sampling schemes, because it is heavily influenced by the number of samples analyzed from each gene pool.

Furthermore, the PCA plot showed that Test panel UK clearly deviated from the two other populations. However, as five individuals were present in both Test panel DK and Test panel UK, and these were found to be located in different positions in the PCA plots, some experimental bias in the determination of genetic markers was, at least partly, responsible for the population grouping. The genotype data in this study was originally filtered so that markers with more than 50% missing data were removed, and only individuals with less than 70% missing markers were retained. However, this means that two random individuals could in principle have been represented by two entirely different marker sets. Indeed, when using a much more stringent filter, where markers had less than 1% missing data and each individual contained less than 10% missing markers, the PCA plot changed significantly, in which the two test panels overlap the MASPOT populations, while still retaining a clear grouping of each panel. The inconsistency of markers from one panel to another is a major challenge using GBS, since the marker selection is random, influenced by small differences in restriction enzyme digestion and DNA molecules sampled for sequencing. Each time new individuals are added to a training population, a number of marker sites are not observed. Consequently, if very large genotyping populations are used, very few, if any, markers will be observed in all samples. Hence, a logical consequence of the present study given that fewer markers retain predictive power is to use more restrictive restriction enzyme combinations to generate fewer genotyping sites. This will decrease the number of potential markers in the analysis, but increase the number of markers common to all, or nearly all, samples and likely benefit the analysis.

### Genomic prediction models

All 167,637 markers were used to develop genomic prediction models with GBLUP. It has been suggested that Bayesian models are more suitable when the training population and the prediction population are genetically distant (Habier et al., 2007, 2013), and especially when the size of the training population is small (Onogi et al., 2014). However, in this study, predictions conducted with BayesA and BayesC (see Supplementary Tables S2, S3) performed equally to predictions with GBLUP, even across populations.

In general, good predictions of chipping quality appeared to be difficult to obtain, especially across populations, but also within-population for Test panel DK. Predictions of dry matter content were more robust both within and across populations. However, Test panel DK did not predict GEBVs in Test panel UK or MASPOT well for either trait. This was probably due to the (too) small panel size of Test panel DK. It did not contain a sufficient number of individuals to represent the diversity of genotypes present in the other two populations sufficiently well. Obviously, the lack of important genotypes (variables) in the model will lead to poor prediction ability. Despite of this, surprisingly good prediction correlation was observed with Test panel DK as training population when predicting dry matter content for the MASPOT population. This was particularly peculiar since the phenotypic profile for dry matter was quite different for each panel, and Test panel DK was the most extreme. Test panel DK contained the 18 parents that were used to generate the MASPOT population. The parents were thus well represented marker-wise in the MASPOT population as well and the genetic variation within the MASPOT population was also present in Test Panel DK. Consequently, the power to predict Test Panel DK may be overestimated because the parents of MASPOT are extremely well characterized and thus inflates the overall estimate. For chipping quality, however, this effect was not observed, though this might be caused by the small size of Test panel DK, as only 39 individuals had chipping quality data available (10 parents of MASPOT). In fact, with such a small panel size, Test panel DK is likely too small to be used as a training population altogether, since prediction accuracy obviously is highly dependent on training population size (Lorenz et al., 2011). When using subsets of either 39 or 80 individuals of each population, the dependency was also apparent, as prediction correlations were generally substantially lower compared to predictions made with the larger and more appropriately sized training populations. Arruda et al. (2015) found that prediction accuracy in wheat increased by 11.2% when increasing the training population from 96 to 144, while reaching a plateau between 192 and 218 individuals. Lorenzana and Bernardo (2009) found an increase in prediction accuracy in maize up to 32% when increasing the training population from 96 to 178, and while they found good prediction accuracies in training populations with only 48 individuals, this varied between traits.

In a previous study where GS models were generated for chipping quality and starch content using the MASPOT population and a test panel similar to Test panel DK, prediction accuracies for the test panel when using the MASPOT population as training population were considerably lower compared to results obtained in the present study (Sverrisdóttir et al., 2017). The test panel used in the previous study was the same panel as Test panel DK in this study with the exception of the parents to the MASPOT population, which likely explains the increase in cross population prediction accuracy observed in this study. Indeed, this also underscores that including a related subpopulation (the MASPOT parents) unproportionally increases prediction accuracy as observed by others (Gowda et al., 2014). Obviously, this represents inflated prediction estimates compared to what can be expected from applying the obtained model to unrelated material.

Test panel UK could predict dry matter in the MASPOT population with good accuracy, but failed to predict chipping quality, and the same was also true the other way around. This may be caused by the fact that chipping quality generally is the more difficult trait to predict either because the environment has more impact on heritability of chipping quality than for dry matter content, or because the number of complex genetic interactions, such as epistasis which are not accounted for in the models, is more pronounced for chipping quality than for dry matter content. In principle, it could also stem from the fact that different non-overlapping sets of alleles are important for this trait in the two panels. This may be unlikely, but cannot be addressed in detail with the current data. In fact, this lower prediction ability across populations is not uncommon and was expected. It is similar to cross-breed predictions in livestock (De Roos et al., 2009; Iheshiulor et al., 2016) and maize (Albrecht et al., 2011; Windhausen et al., 2012), where comparable decreases in prediction accuracies have been observed.

Highly interesting, prediction correlations obtained with the combined population were roughly the same as the prediction correlations obtained with within-population models, indicating that the contributing factor to good predictions with the combined population were the other individuals of the same population and that other populations can be subsequently added to generate models of broader applicability without compromising prediction accuracy. Also, the increase of the population size in the combined population did not have any significant impact on predictions in general, suggesting that the genotype-phenotype data within each population provides the same degree of useful information as the combined population, and hence maximally predicted in the within-population models. In other words, including more individuals in general to the model will not increase prediction accuracy *per se*. What seems to be of greater importance is the composition of the population, and to achieve a prediction model with wider applicability, it is not important to include more individuals to more precisely estimate the effect of alleles already existing in the population, but to include individuals containing new genotypes and hence estimate the effect of alleles not accounted for in the existing model. This is similar findings observed in dairy cattle (De Roos et al., 2009).

Even though the prediction correlations were roughly the same between within-population predictions and predictions with the combined population, the bias was somewhat different for all populations. For dry matter predictions, prediction bias was smaller when using the combined population compared to within-population predictions, i.e. slopes closer to 1, while the opposite was true for chipping quality predictions. Overall, the general tendency for the prediction models was rather large biases above 1. Biases above 1 means that the scale of the predicted values is deflated relative to their observed phenotypes, such that the lowest values are overestimated and vice versa. Very large biases may actually be the result if all the individuals are predicted to have roughly the same value, even though the observed values vary. This is indicative of having no predictive ability since the correlation would merely reflect the observed phenotypic variance rather than the prediction accuracy of the GEBVs. This is in fact the case for when the Test panel DK was used to predict the individuals in Test panel UK. In contrast, predictions done within the MASPOT population had small biases between 1.04 and 1.09, and in most cases, also the combined population. These differences could be caused by a number of factors. For one, the MASPOT population was significantly larger than both Test panel UK and Test panel DK, potentially giving rise to more robust prediction models with respect to bias. In addition, as seen in the PCA, the genetic diversity is large within the MASPOT population, while the diversity in the test panels is restricted by selection through breeding. Although the population size should be factored in, the results also might suggest that an unselected population such as the MASPOT population gives a more robust prediction model compared to panels that have been subjected to selection such as the two test panels. Indeed, the study of Zhao et al. (2012) showed a similar effect in maize, where substantially lower prediction accuracies were obtained when using populations where only the highest genotypic values were selected, compared to prediction accuracies obtained in unselected populations of the same size. Furthermore, in a breeding program, it is just as important to deselect poor performers as to select good breeding candidates, and therefore it is essential to create a training population that captures a broad range of phenotypes.

### Marker number

Surprisingly, reducing the marker set from 167,637 markers to only 7,800 markers did not have any considerable effect on the prediction accuracies, whether the markers were selected to reduce the number of missing markers significantly, or if they were randomly selected, retaining a high proportion of missing data. This suggests that only a small fraction of the 167,637 markers were used for prediction, making the abundance of markers redundant. Indeed, although a higher marker density would be beneficial in theory, in practice, prediction accuracy usually reaches a plateau with increased marker number (Lorenz et al., 2012; Heslot et al., 2015). Studies have shown that increasing the number of markers has conflicting effects on accuracy (Muir, 2007; Meuwissen, 2009; Lorenz et al., 2011). Increased marker density has the clear benefit of being able to capture more of the genetic variance, potentially leading to better predictions and higher accuracies. However, increased marker density also means increased collinearity between markers, which has been found to produce overfitted models with reduced prediction accuracy (Muir, 2007; Shengqiang et al., 2009; Grenier et al., 2015). It has been suggested that in order to make use of increased marker density, the training population size must be scaled with marker numbers to successfully capture the additional information provided by increased marker density, otherwise any positive effect on accuracy from increasing marker numbers can be constrained by the training population size (Muir, 2007; Meuwissen, 2009; Lorenz et al., 2011).

Although in general, there was no considerable difference between predictions done with the 7,800 cherry-picked markers or the 7,800 randomly chosen markers, there were exceptions for predictions of Test panel UK, where lower prediction accuracies were obtained when using the random 7,800 marker set. We speculate that the reason for this is that the number of markers carrying useful information among the sampled 7,800 markers is higher for the MASPOT population and the related Test Panel DK population, than for the Test Panel UK population, because more individuals were sampled in MASPOT than in Test Panel UK. Consequently, the chance of a marker carrying functional information to be included in the random sample is larger for MASPOT than for Test Panel UK. Indeed, further reducing the number of markers sampled leads to decreasing prediction accuracy also for the MASPOT population (data not shown). In support of this is the observed high variance of the prediction accuracies between the different sampling of the random 7,800 markers, indicating that the 7,800 markers are close to the threshold of the minimum numbers of markers necessary to make a prediction model with any predictive power at all.

As mentioned, there was a large number of missing data in the used marker set of 167,637, which is very common for GBS data. For that reason, imputation is a necessary part of GBS data processing. In this study, mean imputation was used, where missing values were replaced by the mean of non-missing values. Mean imputation is one of the simplest imputation methods, and it requires a minimum of computational time. Studies have shown that it performs equally well to other more demanding imputation methods with regards to prediction accuracy (Poland et al., 2012; Arruda et al., 2015). However, Poland et al. (2012) showed a significant difference between imputation methods in terms of marker imputation error, in which mean imputation generated some of the highest errors. They also showed that even though the imputation method did not have an effect on the prediction accuracy, significant biases in the GEBVs were observed when using mean imputation in contrast to imputation methods such as multivariate-normal expectation-maximization algorithm. In addition, Rutkoski et al. (2013) showed that the difference in performance among imputation methods is increased as the number of missing data increases, and at high levels of missing data (70%), mean imputation performs significantly worse. In this study, markers for the larger data set of 167,637 markers were filtered to remove missing data above 50%. With this relatively high threshold other imputation methods might be better fitted. However, compared to the smaller data set, where markers with missing data above 10% were removed, no significant changes were observed. Since there were no significant differences in prediction accuracies between the full marker set of 167,637 markers and the reduced sets of 7,800 markers selected randomly or cherry-picked, this suggests that in neither case the model is constrained by the number of genotyping sites. However, in most cases the bias was improved when using the smaller marker sets, particularly the 7,800 filtered and selected markers. This is probably caused by an overfitting of collinear markers in prediction models when using the larger marker set.

## Conclusions

The main aims of this study were to study genomic predictions of chipping quality and dry matter across potato breeding populations, to examine if prediction accuracy could be increased by expanding the training population, and finally, to study the effect of marker density on predictions. Expanding the training population by combining all three populations did in general not generate a gain in prediction accuracy, as predictions for each model gave the same results whether using the combined population or predicting within-population. Similar to several studies in plants and animals, we mainly obtained low or moderate prediction correlations across populations, and in all cases the biases were large, meaning that the scale of predicted values was deflated, or in a few cases, inflated, relative to the observed phenotypic values. However, the combined population had high prediction accuracy for all populations simultaneously, and thus could be applied to all populations. This suggests that it is indeed possible to obtain a general potato prediction model if all relevant genotypes are included in the model and furthermore, this can be done in an iterative fashion including several studies.

## Author contributions

ES and KN designed the research and wrote the manuscript; ES performed the genotyping experiments and analyzed the data; TA contributed to GBS data processing; ERS and HJ contributed to the phenotyping of the MASPOT population; HK created mapping population at breeding station, provided plant material and performed field experiments; LJ contributed to GBS data analysis; GB provided genetic material and phenotypic data of Test panel UK.

### Conflict of interest statement

HK is employed by the commercial breeding and manufacturing company Danespo. The remaining authors declare that the research was conducted in the absence of any commercial or financial relationships that could be construed as a potential conflict of interest.
