# Supplementary material for: The Value of Expanding the Training Population to Improve Genomic Selection Models in Tetraploid Potato
Source: Front Plant Sci. 2018 Aug 6;9:1118. doi: 10.3389/fpls.2018.01118 (PMC6090097; doi:10.3389/fpls.2018.01118)
Supplement: Supplementary file 2 [file Data_Sheet_2.DOCX]

Supplementary Material

The value of expanding the training population in genomic selection models for tetraploid potato

Elsa Sverrisdóttir*, Ea Høegh Riis Sundmark, Heidi Øllegaard Johnsen, Hanne Grethe Kirk, Torben Asp, Luc Janss, Glenn Bryan, and Kåre L. Nielsen

*** Correspondence:** Elsa Sverrisdóttir: esv@bio.aau.dk

# Supplementary File 2: List of cultivars in Test panel DK

The following is a list of the cultivars and breeding clones selected for Test panel DK in the genomic selection project. The cultivars were grown, harvested and phenotyped over a number of years at the breeding station in Vandel, Denmark. The phenotypic data for chipping quality and dry matter content is listed as the mean over several years and replicates. The 18 cultivars that were used as parents to generate the MASPOT population are marked with bold lettering.

| Cultivar | Chipping quality [scale 1-9] | Dry matter content [%] |
| --- | --- | --- |
| 00-DLS-1 | 5.5 | 27.99 |
| 01-EAS-1 | NA | 28.82 |
| 01-EAZ-4 | NA | 28.18 |
| 01-EBQ-9 | NA | 26.45 |
| 02-EMC-9 | NA | 27.73 |
| 02-ENU-5 | 6 | 28.63 |
| 03-GAE-4 | NA | 28.31 |
| 05-DLG-26 | NA | 26.31 |
| 05-GUK-3 | 5.5 | 23.55 |
| 05-GUR-1 | 4.5 | 27.11 |
| 06-ECI-7 | NA | 28.10 |
| 06-ELB-14 | NA | 26.46 |
| 06-LDP-1 | 4.5 | 27.52 |
| 06-LEE-4 | 4 | 27.94 |
| 06-LEK-4 | 4.5 | 28.35 |
| 07-LPL-3 | NA | 27.19 |
| 07-LPW-4 | 5.67 | 28.04 |
| 08-GJY-24 | 4.5 | 28.32 |
| 08-GPZ-5 | NA | 27.87 |
| 08-LWK-1 | NA | 29.60 |
| 08-LWZ-1 | NA | 28.81 |
| 08-LXB-1 | NA | 26.57 |
| 08-LXB-10 | NA | 27.55 |
| 08-LXC-3 | NA | 27.35 |
| 08-LXC-4 | NA | 26.76 |
| 08-LXD-2 | NA | 27.40 |
| 08-LXE-2 | NA | 27.48 |
| 08-LXJ-5 | NA | 29.04 |
| 08-LZH-3 | NA | 26.29 |
| 08-LZK-1 | 6 | 26.82 |
| 08-MAK-06 | 3 | 26.40 |
| 08-MAK-07 | 3 | 24.84 |
| 08-MAW-02 | NA | 27.33 |
| 09-0-192-04 | 4.33 | 23.36 |
| 09-GUT-23 | 2 | 29.22 |
| 09-GUT-25 | NA | 28.94 |
| 09-LPV-6 | NA | 27.75 |
| 09-MDI-7 | NA | 27.01 |
| 09-MDP-12 | NA | 27.79 |
| 09-MDP-4 | NA | 27.07 |
| 09-MDP-5 | NA | 28.04 |
| 09-MDP-6 | NA | 27.50 |
| 09-MDP-9 | NA | 28.87 |
| 09-MDR-4 | 5 | 28.25 |
| 09-MFD-6 | 5 | 26.75 |
| 89-ZAM-16 | 2.83 | 22.90 |
| 92-BPU-30 | 3 | 24.10 |
| 93-CAG-1 | 4 | 26.88 |
| 93-CAL-3 | 3.5 | 25.26 |
| 96-BQD-56 | NA | 27.76 |
| Artana | NA | 26.16 |
| Bonanza | 6 | 25.41 |
| Bruse | 4 | 23.87 |
| **07-LJE-1** | **7.5** | **26.67** |
| **04-GIV-03** | **NA** | **25.75** |
| **05-GQE-02** | **NA** | **23.29** |
| **07-LIX-5** | **NA** | **22.17** |
| **89-BJQ-4** | **4.5** | **22.20** |
| **93-CAQ-14** | **2** | **22.93** |
| **96-BYM-8** | **2** | **18.58** |
| **Agria** | **5.25** | **21.16** |
| **Aventra** | **NA** | **22.46** |
| **Desiree** | **3.2** | **21.82** |
| **Florice** | **NA** | **17.02** |
| **Jutlandia** | **3.31** | **20.26** |
| **Kuras** | **3.8** | **26.42** |
| **Rywal** | **NA** | **21.85** |
| **Sarpo Mira** | **NA** | **22.99** |
| **Shepody** | **2** | **21.92** |
| **Spunta** | **3.48** | **19.43** |
| **Isle of Jura** | **NA** | **20.57** |
| Camel | 2.25 | 17.56 |
| Canasta | 5 | 26.73 |
| Centaure | NA | 23.90 |
