# Supplementary material for: The Value of Expanding the Training Population to Improve Genomic Selection Models in Tetraploid Potato
Source: Front Plant Sci. 2018 Aug 6;9:1118. doi: 10.3389/fpls.2018.01118 (PMC6090097; doi:10.3389/fpls.2018.01118)
Supplement: Supplementary file 3 [file Data_Sheet_3.DOCX]

Supplementary Material

The value of expanding the training population in genomic selection models for tetraploid potato

Elsa Sverrisdóttir*, Ea Høegh Riis Sundmark, Heidi Øllegaard Johnsen, Hanne Grethe Kirk, Torben Asp, Luc Janss, Glenn Bryan, and Kåre L. Nielsen

*** Correspondence:** Elsa Sverrisdóttir: esv@bio.aau.dk

# Supplementary File 3: Adapter and primer sequences

*Underlined sequences represent indexes.*

5’ Index adapter

1. 5’-ACACTCTTTCCCTACACGACGCTCTTCCGATCTTGCA
2. 5’-ACACTCTTTCCCTACACGACGCTCTTCCGATCTTGCGA
3. 5’-ACACTCTTTCCCTACACGACGCTCTTCCGATCTCGCTT
4. 5’-ACACTCTTTCCCTACACGACGCTCTTCCGATCTGGTTGT
5. 5’-ACACTCTTTCCCTACACGACGCTCTTCCGATCTCCAGCT
6. 5’-ACACTCTTTCCCTACACGACGCTCTTCCGATCTTATTTTT
7. 5’-ACACTCTTTCCCTACACGACGCTCTTCCGATCTCTTGCTT
8. 5’-ACACTCTTTCCCTACACGACGCTCTTCCGATCTACGACTAC

5’ Index adapter reverse complement

1. 5’-CWGTGCAAGATCGGAAGAGCGTCGTGTAGGGAAAGAGTGT
2. 5’-CWGTCGCAAGATCGGAAGAGCGTCGTGTAGGGAAAGAGTGT
3. 5’-CWGAAGCGAGATCGGAAGAGCGTCGTGTAGGGAAAGAGTGT
4. 5’-CWGACAACCAGATCGGAAGAGCGTCGTGTAGGGAAAGAGTGT
5. 5’-CWGAGCTGGAGATCGGAAGAGCGTCGTGTAGGGAAAGAGTGT
6. 5’-CWGAAAAATAAGATCGGAAGAGCGTCGTGTAGGGAAAGAGTGT
7. 5’-CWGAAGCAAGAGATCGGAAGAGCGTCGTGTAGGGAAAGAGTGT
8. 5’-CWGGTAGTCGTAGATCGGAAGAGCGTCGTGTAGGGAAAGAGTGT

3’ Index adapter

1. 5’-CWG AGATCGGAAGAGCACACGTCTGAACTCCAGTCACATCACGATCTCGTATGCCGTCTTCTGCTTG
2. 5’-CWG AGATCGGAAGAGCACACGTCTGAACTCCAGTCACCGATGTATCTCGTATGCCGTCTTCTGCTTG
3. 5’-CWG AGATCGGAAGAGCACACGTCTGAACTCCAGTCACTTAGGCATCTCGTATGCCGTCTTCTGCTTG
4. 5’-CWG AGATCGGAAGAGCACACGTCTGAACTCCAGTCACTGACCAATCTCGTATGCCGTCTTCTGCTTG
5. 5’-CWG AGATCGGAAGAGCACACGTCTGAACTCCAGTCACACAGTGATCTCGTATGCCGTCTTCTGCTTG
6. 5’-CWG AGATCGGAAGAGCACACGTCTGAACTCCAGTCACGCCAATATCTCGTATGCCGTCTTCTGCTTG
7. 5’-CWG AGATCGGAAGAGCACACGTCTGAACTCCAGTCACAGTCAAATCTCGTATGCCGTCTTCTGCTTG
8. 5’-CWG AGATCGGAAGAGCACACGTCTGAACTCCAGTCACACTTGAATCTCGTATGCCGTCTTCTGCTTG
9. 5’-CWG AGATCGGAAGAGCACACGTCTGAACTCCAGTCACGATCAGATCTCGTATGCCGTCTTCTGCTTG
10. 5’-CWG AGATCGGAAGAGCACACGTCTGAACTCCAGTCACTAGCTTATCTCGTATGCCGTCTTCTGCTTG
11. 5’-CWG AGATCGGAAGAGCACACGTCTGAACTCCAGTCACGGCTACATCTCGTATGCCGTCTTCTGCTTG
12. 5’-CWG AGATCGGAAGAGCACACGTCTGAACTCCAGTCACCTTGTAATCTCGTATGCCGTCTTCTGCTTG

3’ Index adapter reverse complement

1. 5’-CAAGCAGAAGACGGCATACGAGATCGTGATGTGACTGGAGTTCAGACGTGTGCTCTTCCGATCT
2. 5’-CAAGCAGAAGACGGCATACGAGATACATCGGTGACTGGAGTTCAGACGTGTGCTCTTCCGATCT
3. 5’-CAAGCAGAAGACGGCATACGAGATGCCTAAGTGACTGGAGTTCAGACGTGTGCTCTTCCGATCT
4. 5’-CAAGCAGAAGACGGCATACGAGATTGGTCAGTGACTGGAGTTCAGACGTGTGCTCTTCCGATCT
5. 5’-CAAGCAGAAGACGGCATACGAGATCACTGTGTGACTGGAGTTCAGACGTGTGCTCTTCCGATCT
6. 5’-CAAGCAGAAGACGGCATACGAGATATTGGCGTGACTGGAGTTCAGACGTGTGCTCTTCCGATCT
7. 5’-CAAGCAGAAGACGGCATACGAGATTTGACTGTGACTGGAGTTCAGACGTGTGCTCTTCCGATCT
8. 5’-CAAGCAGAAGACGGCATACGAGATTCAAGTGTGACTGGAGTTCAGACGTGTGCTCTTCCGATCT
9. 5’-CAAGCAGAAGACGGCATACGAGATCTGATCGTGACTGGAGTTCAGACGTGTGCTCTTCCGATCT
10. 5’-CAAGCAGAAGACGGCATACGAGATAAGCTAGTGACTGGAGTTCAGACGTGTGCTCTTCCGATCT
11. 5’-CAAGCAGAAGACGGCATACGAGATGTAGCCGTGACTGGAGTTCAGACGTGTGCTCTTCCGATCT
12. 5’-CAAGCAGAAGACGGCATACGAGATTACAAGGTGACTGGAGTTCAGACGTGTGCTCTTCCGATCT

PCR primer 1

5’-AATGATACGGCGACCACCGAGATCTACACTCTTTCCCTACACGACGCTCTTCCGATCT

PCR primer 2

5’-CAAGCAGAAGACGGCATACGAGAT
