# Supplementary material for: The Value of Expanding the Training Population to Improve Genomic Selection Models in Tetraploid Potato
Source: Front Plant Sci. 2018 Aug 6;9:1118. doi: 10.3389/fpls.2018.01118 (PMC6090097; doi:10.3389/fpls.2018.01118)
Supplement: Supplementary file 7 [file Data_Sheet_7.DOCX]

Supplementary Material

The value of expanding the training population in genomic selection models for tetraploid potato

Elsa Sverrisdóttir*, Ea Høegh Riis Sundmark, Heidi Øllegaard Johnsen, Hanne Grethe Kirk, Torben Asp, Luc Janss, Glenn Bryan, and Kåre L. Nielsen

*** Correspondence:** Elsa Sverrisdóttir: esv@bio.aau.dk

# Supplementary Figures

**
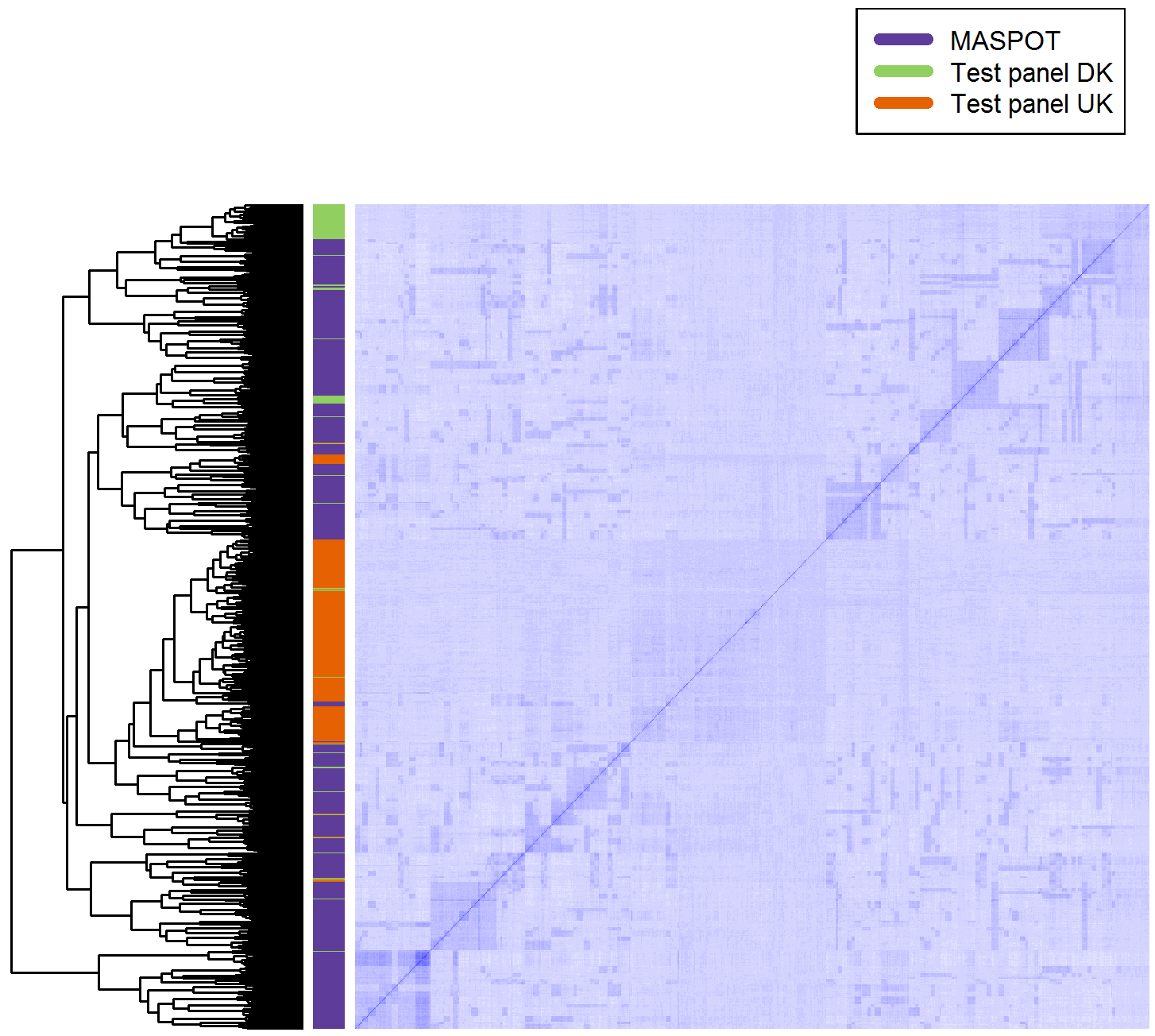
**

**Supplementary Figure S1.** **Heat map of the genomic relationship matrix for the three populations used in the study.** The matrix is obtained from 7,800 markers after stringent filtering for missing data. Rows and columns represent the breeding line. The higher the value and thus the stronger the genetic relationship, the darker the blue color in the heat map is. The colored line next to the dendrogram represent which population the breeding line comes from, as explained in the legend in the top right corner.

**
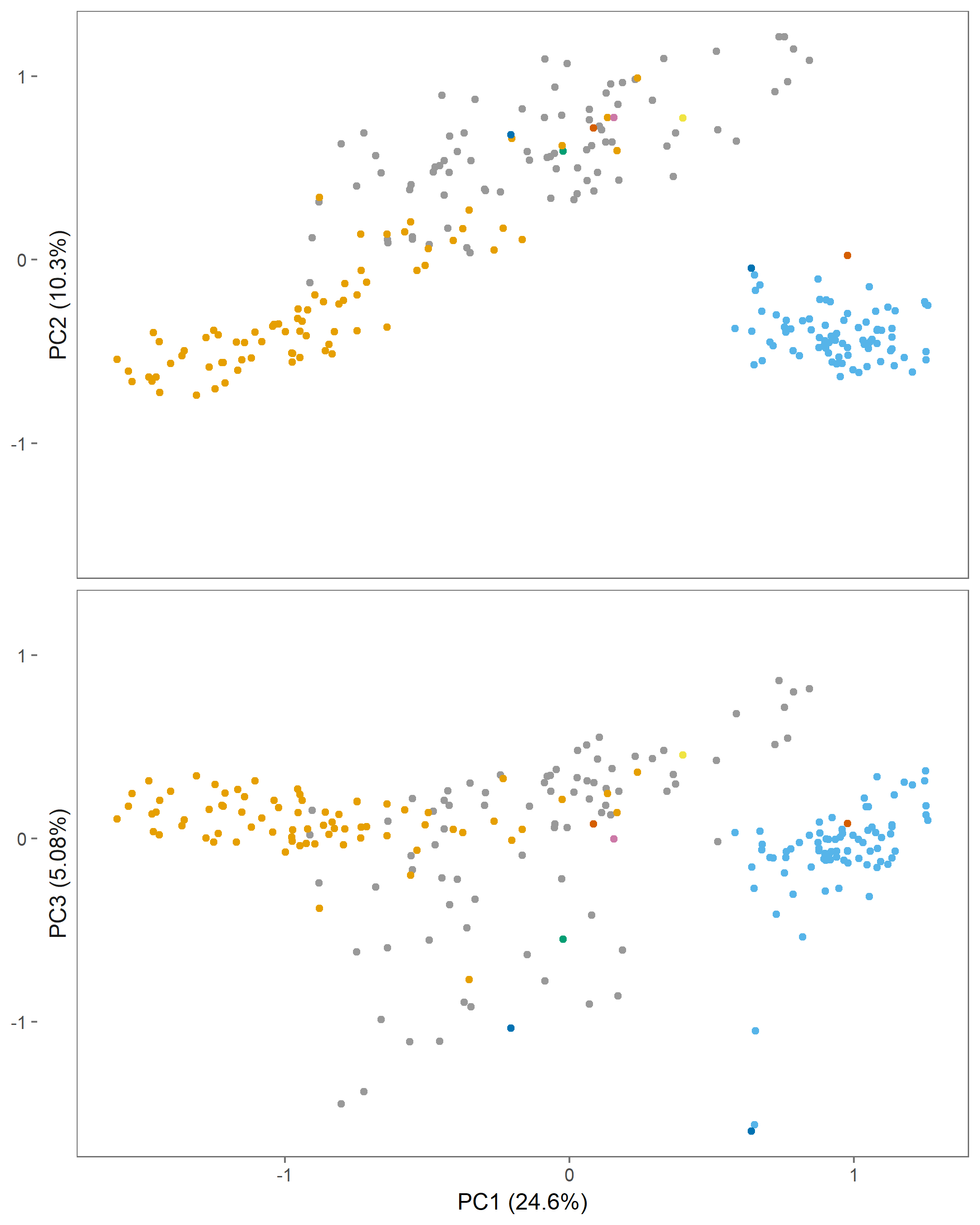
**

**Supplementary Figure S2.** Principal component analysis (PCA) of genomic relationship matrix constructed from genotypes at 167,637 SNP markers using a subset (1) of 80 individuals from each population, MASPOT (grey), Test panel DK (yellow), and Test panel UK (blue). The first principal component (PC1) is plottet against the second principal component (PC2) in the top and against the third principal component (PC3) in the bottom.

**
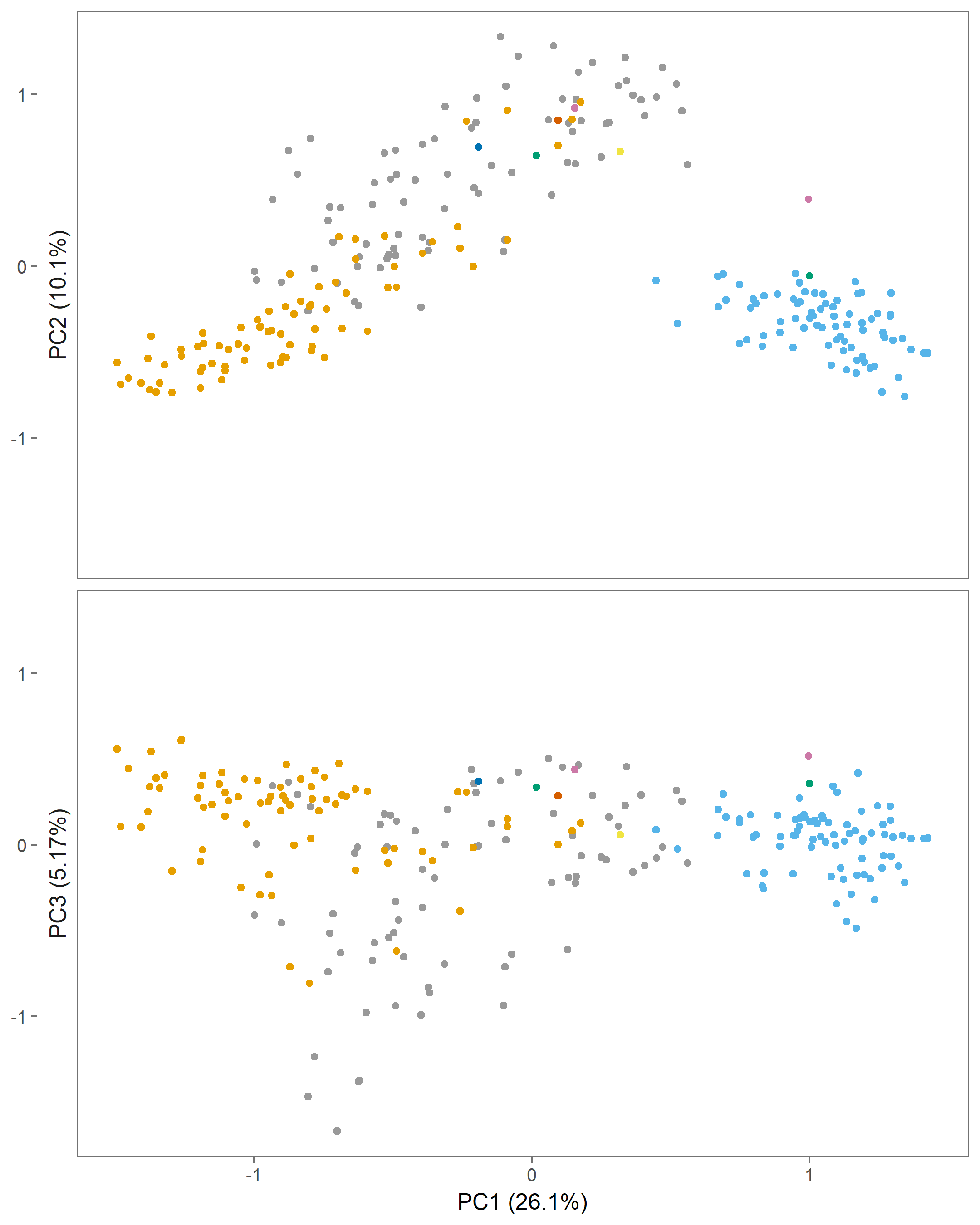
**

**Supplementary Figure S3.** Principal component analysis (PCA) of genomic relationship matrix constructed from genotypes at 167,637 SNP markers using a subset (2) of 80 individuals from each population, MASPOT (grey), Test panel DK (yellow), and Test panel UK (blue). The first principal component (PC1) is plottet against the second principal component (PC2) in the top and against the third principal component (PC3) in the bottom.

**
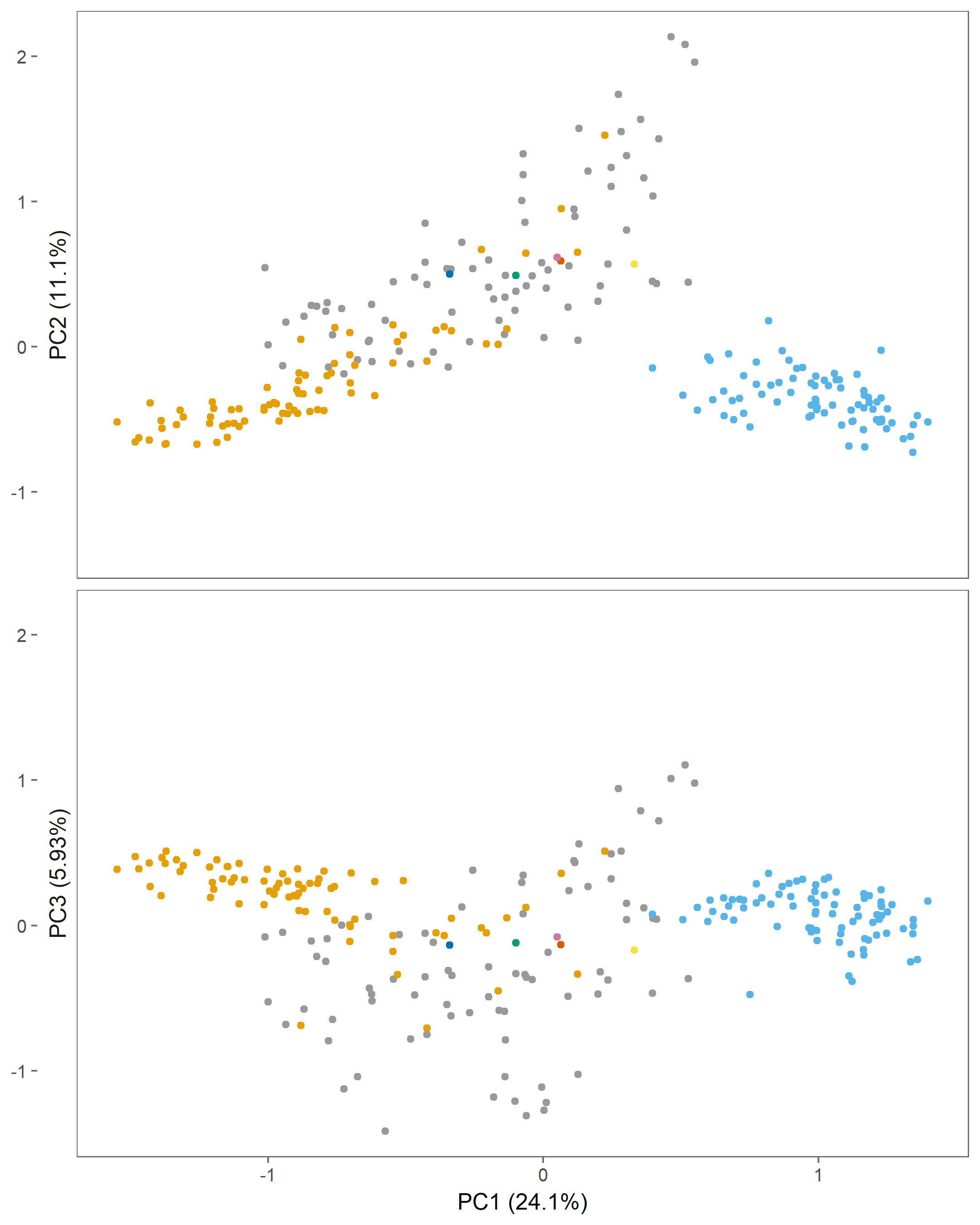
**

**Supplementary Figure S4.** Principal component analysis (PCA) of genomic relationship matrix constructed from genotypes at 167,637 SNP markers using a subset (3) of 80 individuals from each population, MASPOT (grey), Test panel DK (yellow), and Test panel UK (blue). The first principal component (PC1) is plottet against the second principal component (PC2) in the top and against the third principal component (PC3) in the bottom.

**
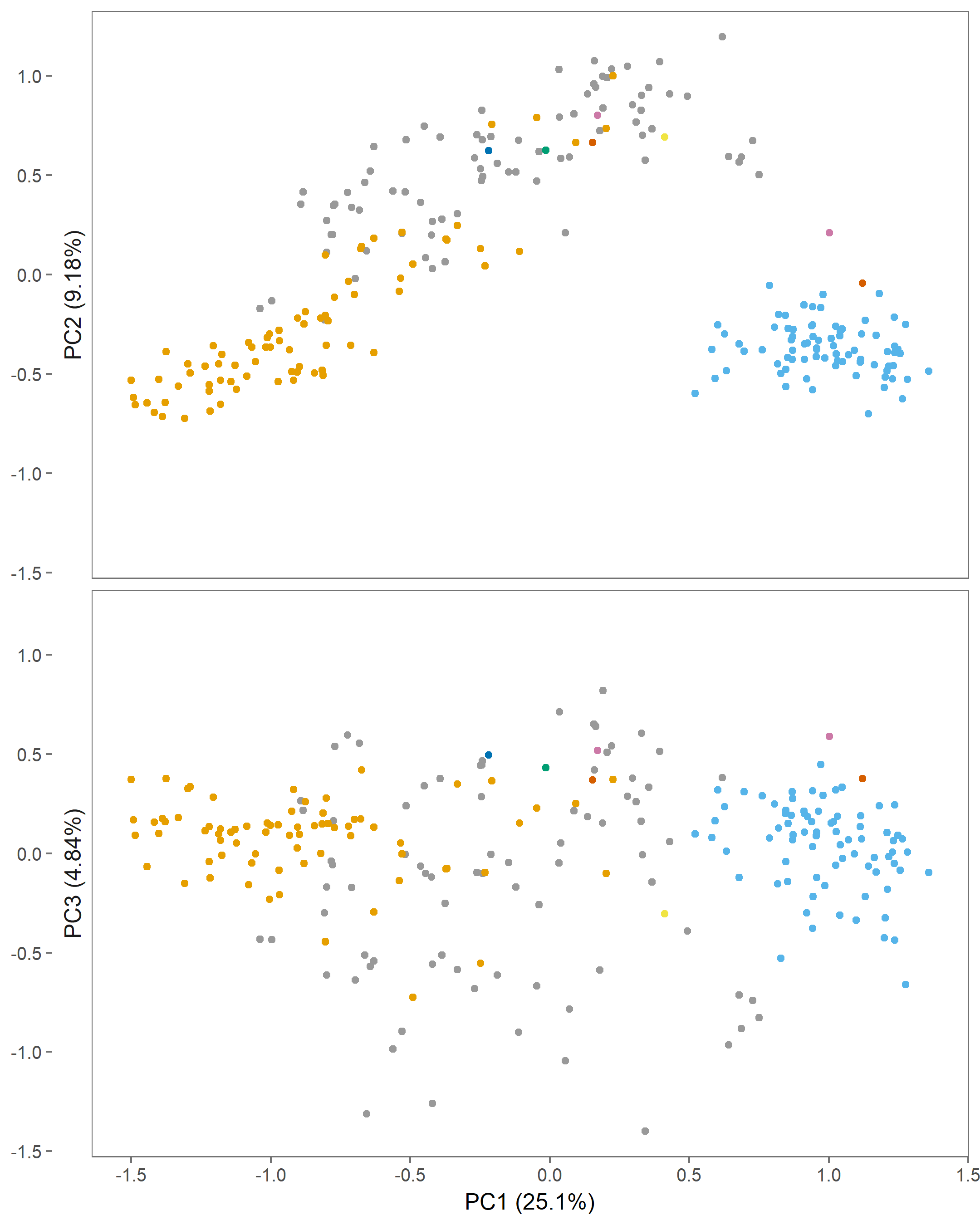
**

**Supplementary Figure S5.** Principal component analysis (PCA) of genomic relationship matrix constructed from genotypes at 167,637 SNP markers using a subset (4) of 80 individuals from each population, MASPOT (grey), Test panel DK (yellow), and Test panel UK (blue). The first principal component (PC1) is plottet against the second principal component (PC2) in the top and against the third principal component (PC3) in the bottom.

**
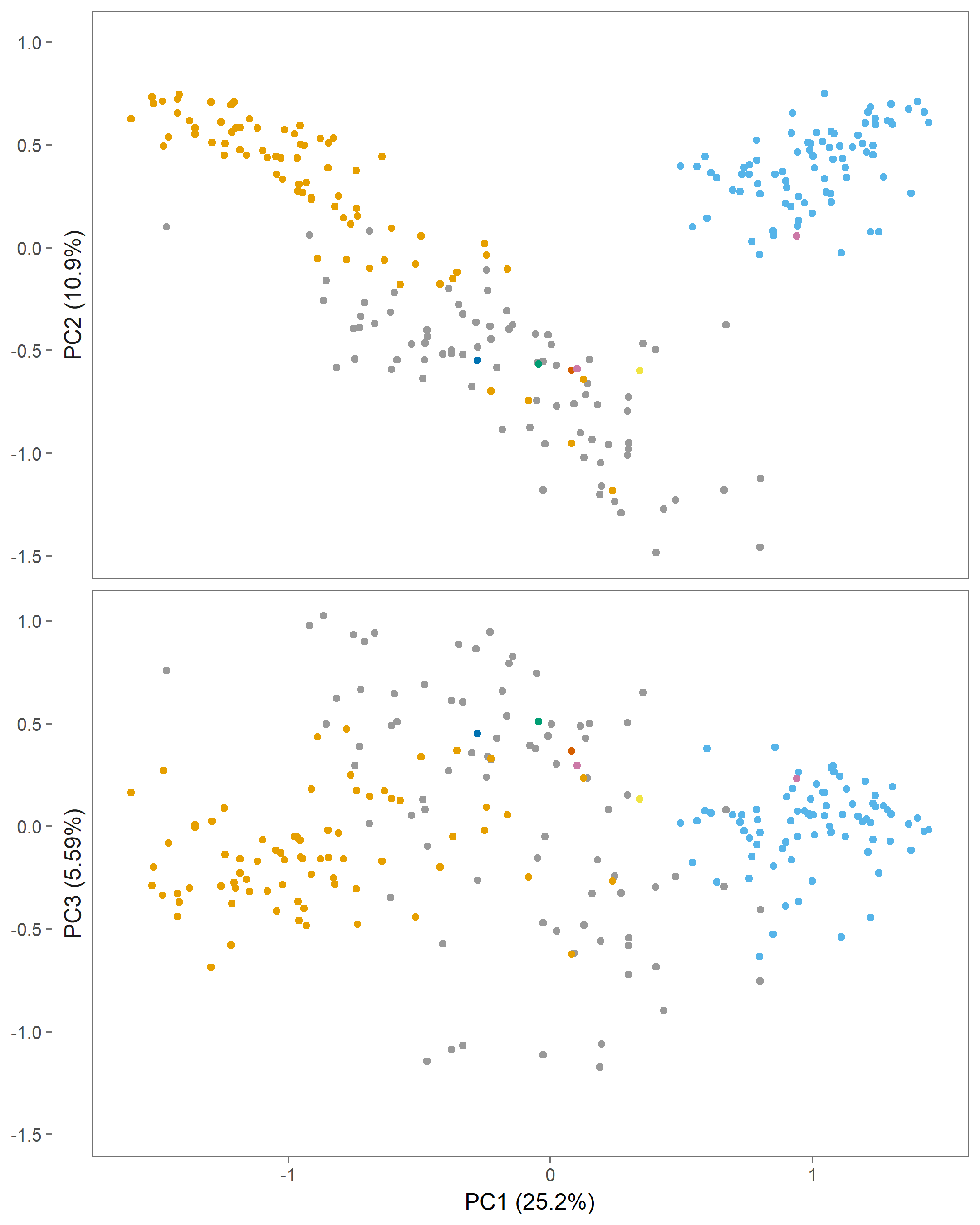
**

**Supplementary Figure S6.** Principal component analysis (PCA) of genomic relationship matrix constructed from genotypes at 167,637 SNP markers using a subset (5) of 80 individuals from each population, MASPOT (grey), Test panel DK (yellow), and Test panel UK (blue). The first principal component (PC1) is plottet against the second principal component (PC2) in the top and against the third principal component (PC3) in the bottom.

**
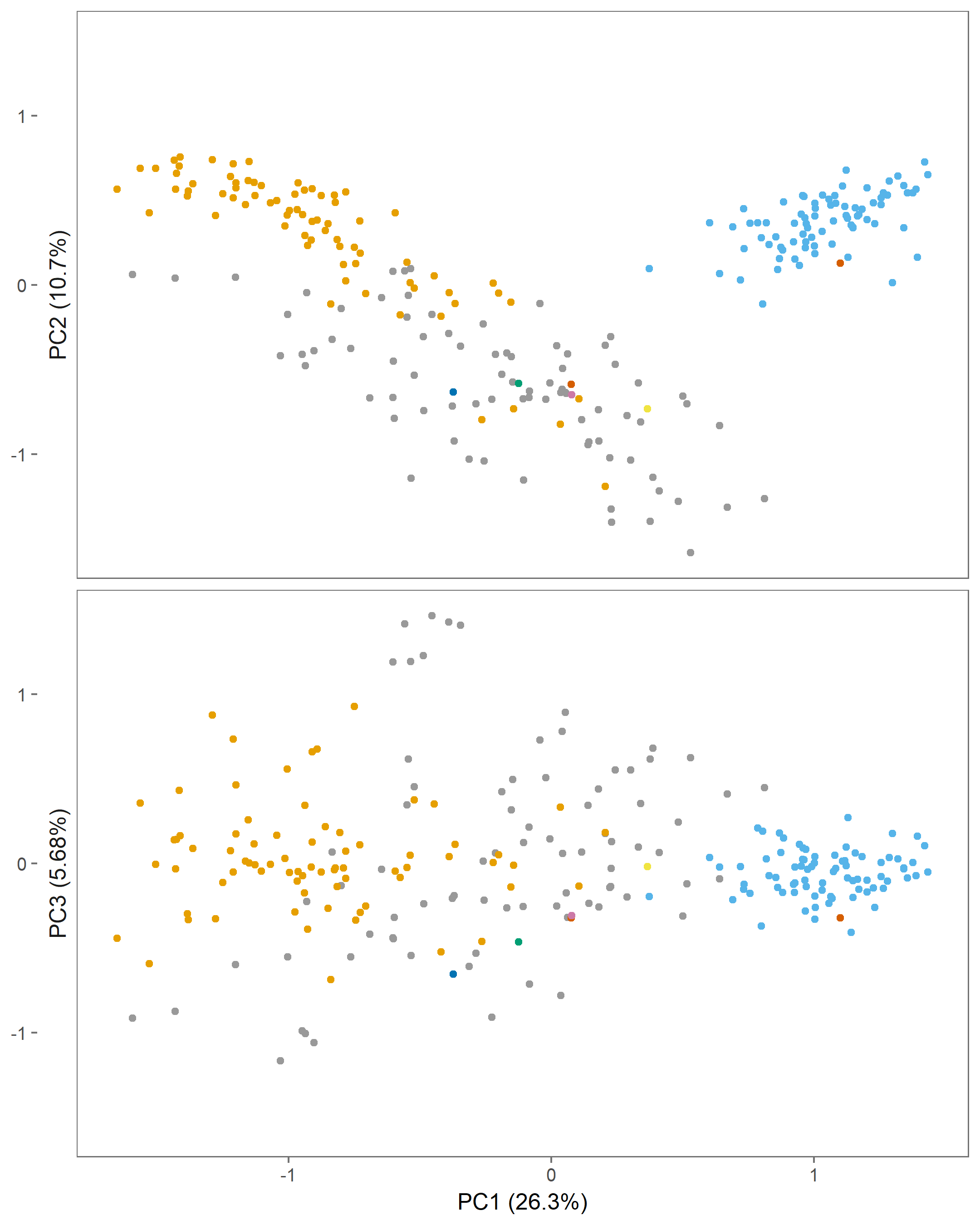
**

**Supplementary Figure S7.** Principal component analysis (PCA) of genomic relationship matrix constructed from genotypes at 167,637 SNP markers using a subset (6) of 80 individuals from each population, MASPOT (grey), Test panel DK (yellow), and Test panel UK (blue). The first principal component (PC1) is plottet against the second principal component (PC2) in the top and against the third principal component (PC3) in the bottom.

**
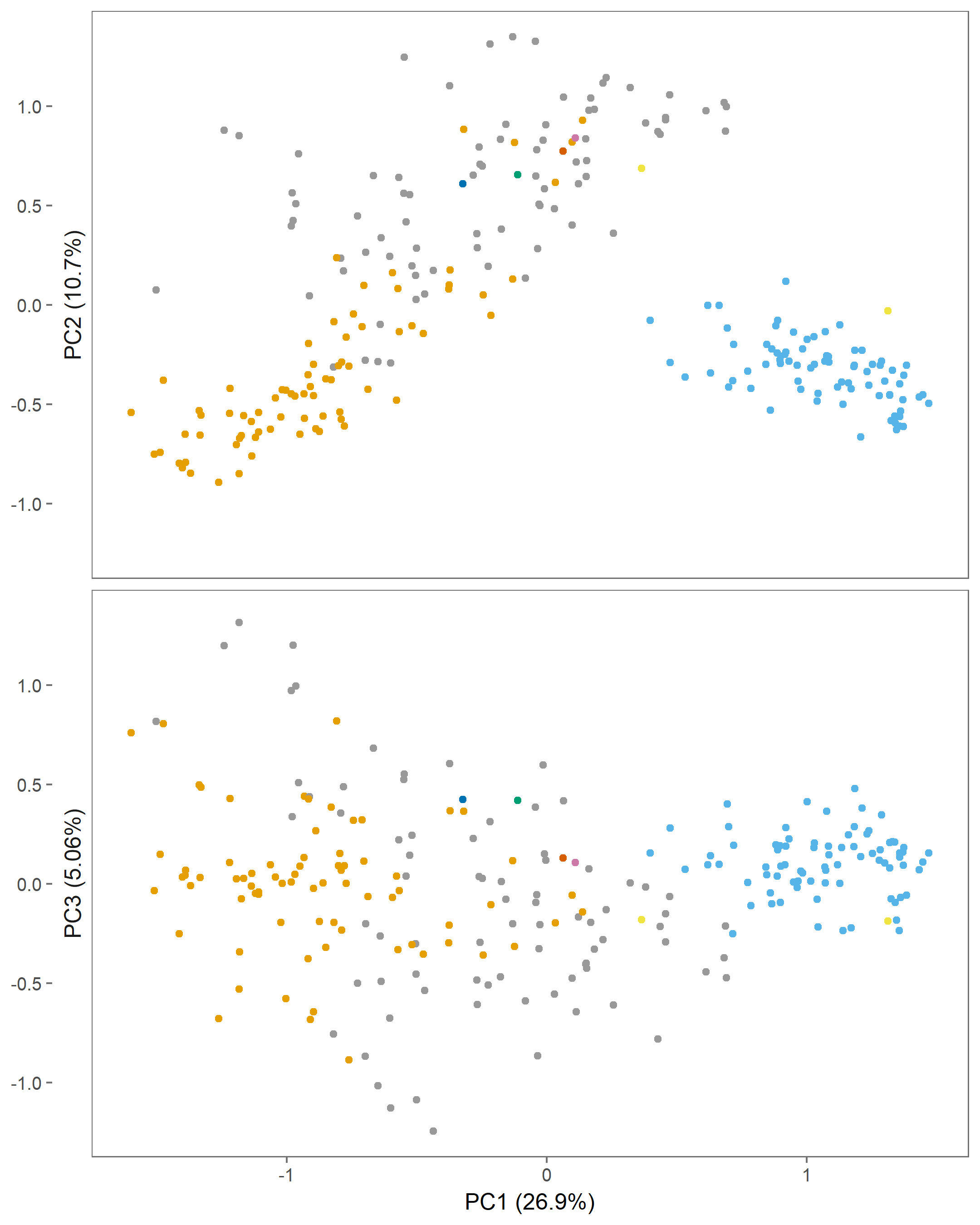
**

**Supplementary Figure S8.** Principal component analysis (PCA) of genomic relationship matrix constructed from genotypes at 167,637 SNP markers using a subset (7) of 80 individuals from each population, MASPOT (grey), Test panel DK (yellow), and Test panel UK (blue). The first principal component (PC1) is plottet against the second principal component (PC2) in the top and against the third principal component (PC3) in the bottom.

**
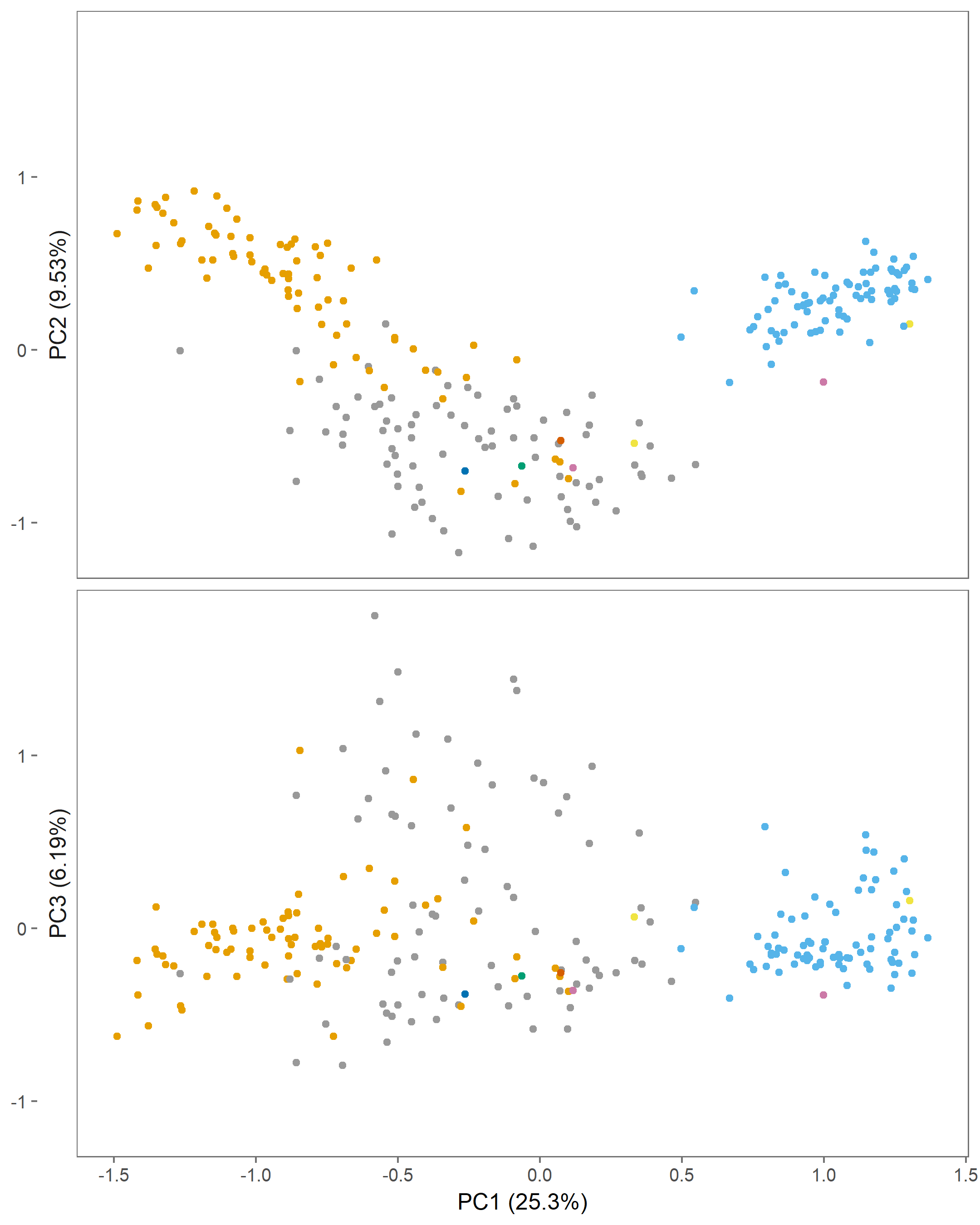
**

**Supplementary Figure S9.** Principal component analysis (PCA) of genomic relationship matrix constructed from genotypes at 167,637 SNP markers using a subset (8) of 80 individuals from each population, MASPOT (grey), Test panel DK (yellow), and Test panel UK (blue). The first principal component (PC1) is plottet against the second principal component (PC2) in the top and against the third principal component (PC3) in the bottom.

**
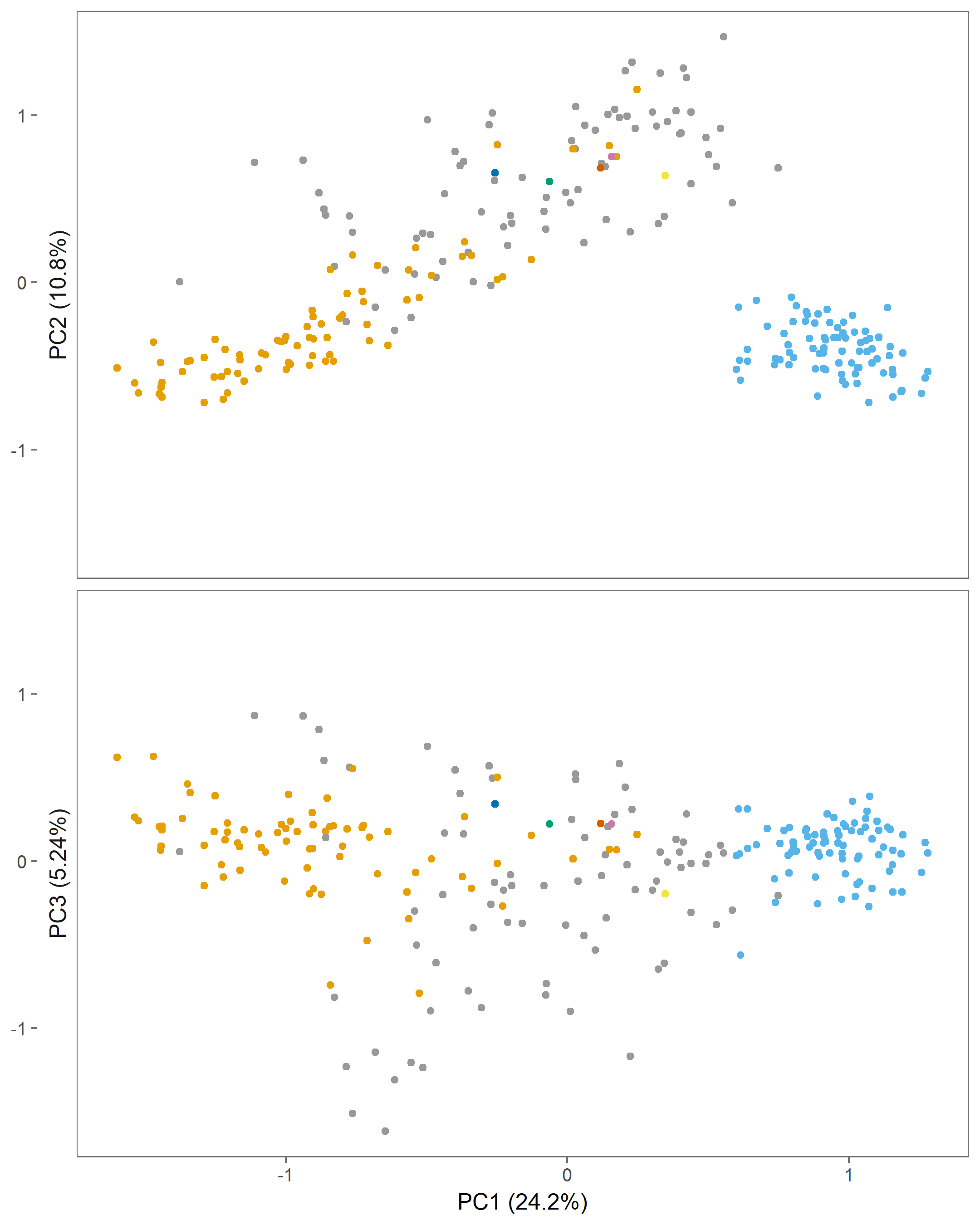
**

**Supplementary Figure S10.** Principal component analysis (PCA) of genomic relationship matrix constructed from genotypes at 167,637 SNP markers using a subset (9) of 80 individuals from each population, MASPOT (grey), Test panel DK (yellow), and Test panel UK (blue). The first principal component (PC1) is plottet against the second principal component (PC2) in the top and against the third principal component (PC3) in the bottom.

**
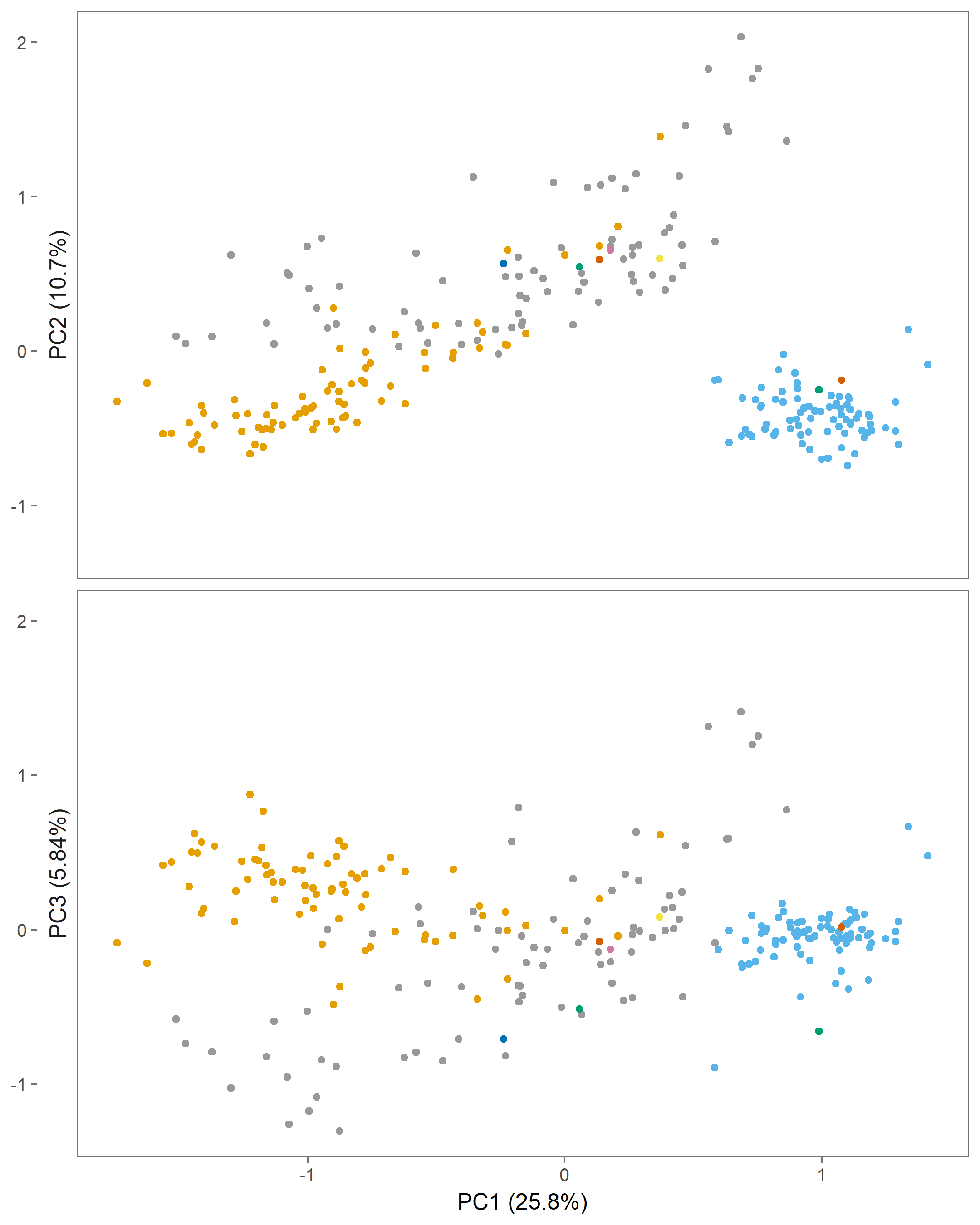
**

**Supplementary Figure S11.** Principal component analysis (PCA) of genomic relationship matrix constructed from genotypes at 167,637 SNP markers using a subset (10) of 80 individuals from each population, MASPOT (grey), Test panel DK (yellow), and Test panel UK (blue). The first principal component (PC1) is plottet against the second principal component (PC2) in the top and against the third principal component (PC3) in the bottom.

**
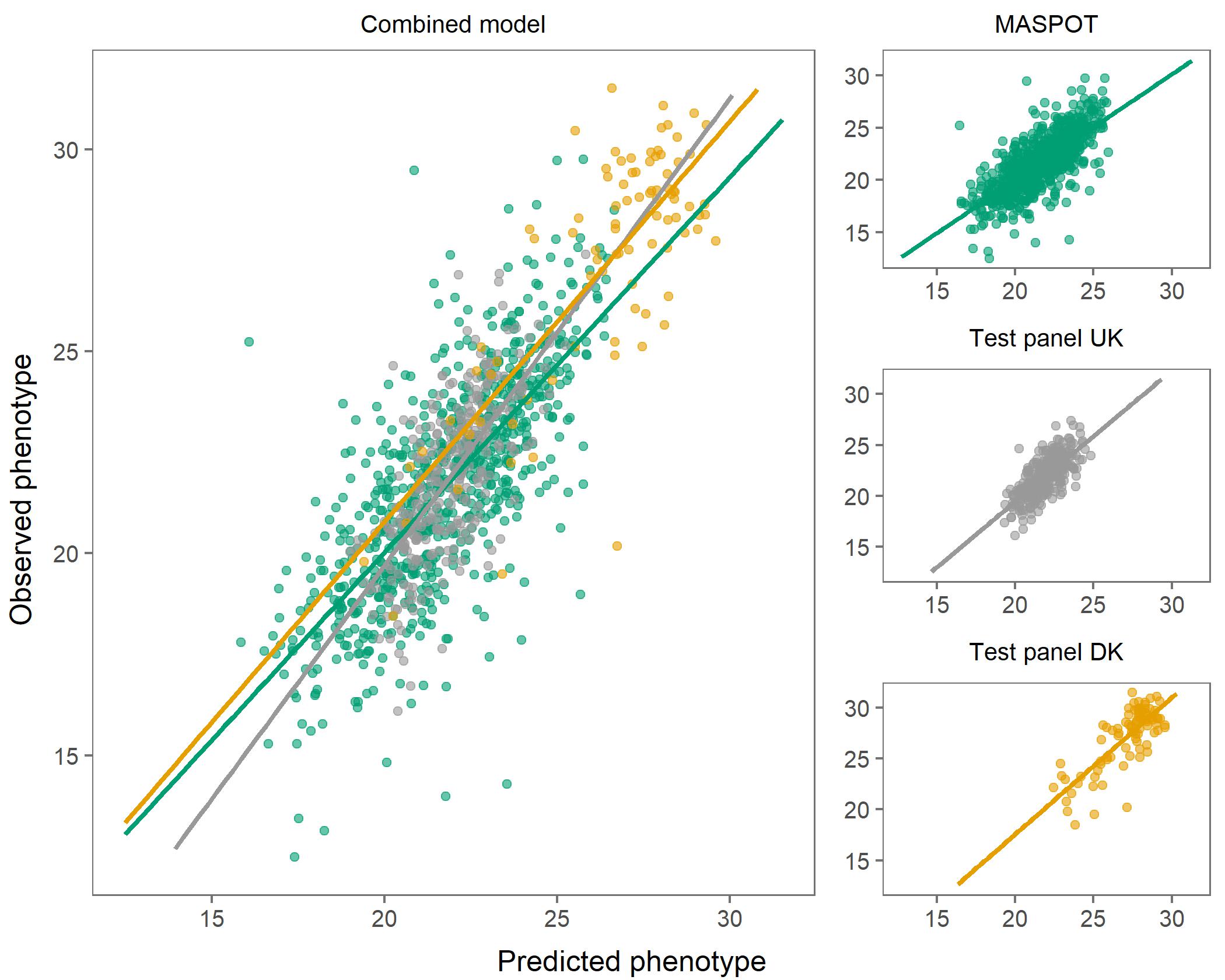
**

**Supplementary Figure S12.** **Predictions of dry matter content with 7,800 cherry-picked markers.** Predictions were made using the combined model (left) or using within-population predictions (right). Green: Predictions of MASPOT population. Grey: Predictions of Test panel UK. Yellow: Predictions of Test panel DK.

**
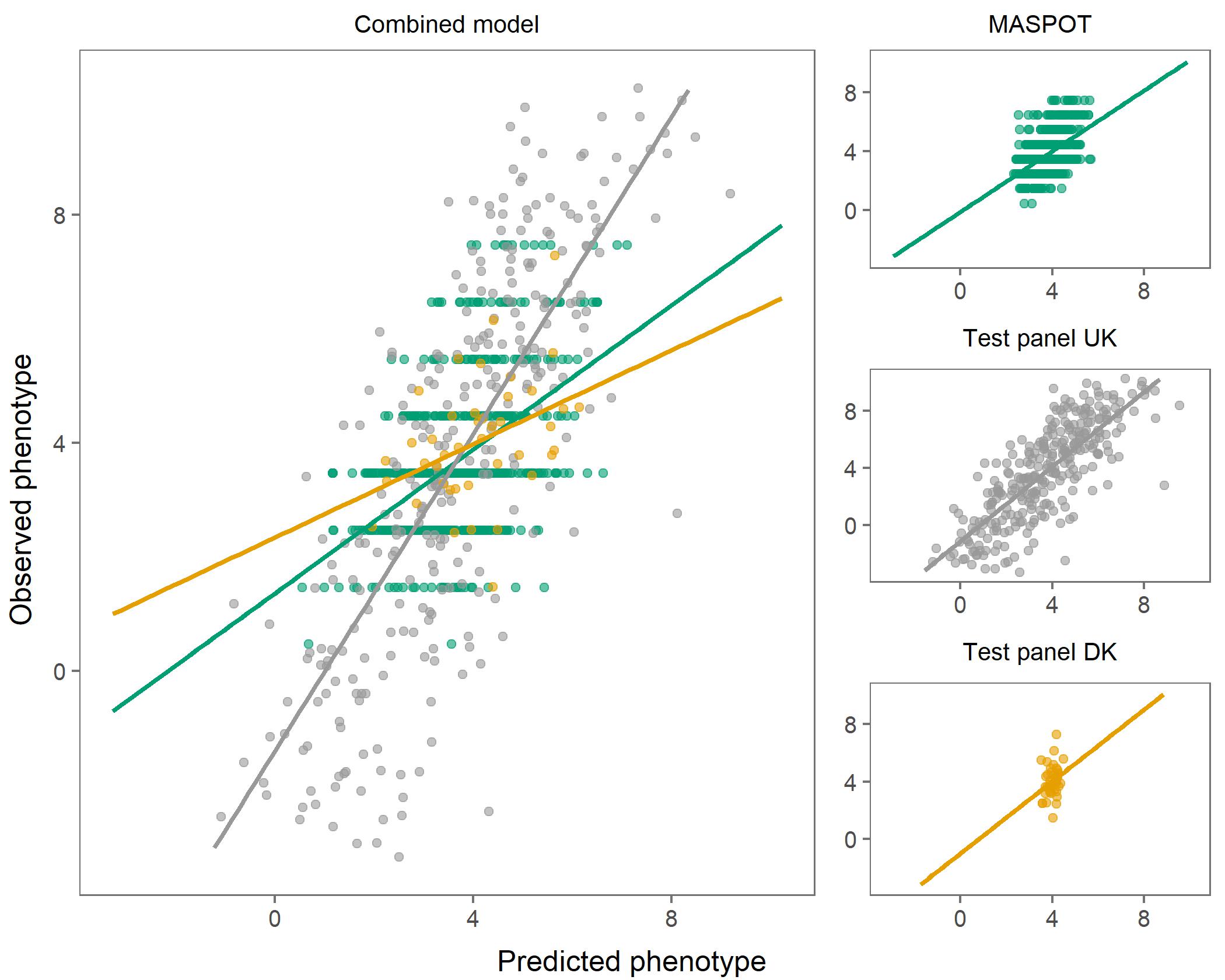
**

**Supplementary Figure S13.** **Predictions of chipping quality with 7,800 cherry-picked markers.** Predictions were made using the combined model (left) or using within-population predictions (right). Green: Predictions of MASPOT population. Grey: Predictions of Test panel UK. Yellow: Predictions of Test panel DK.

**
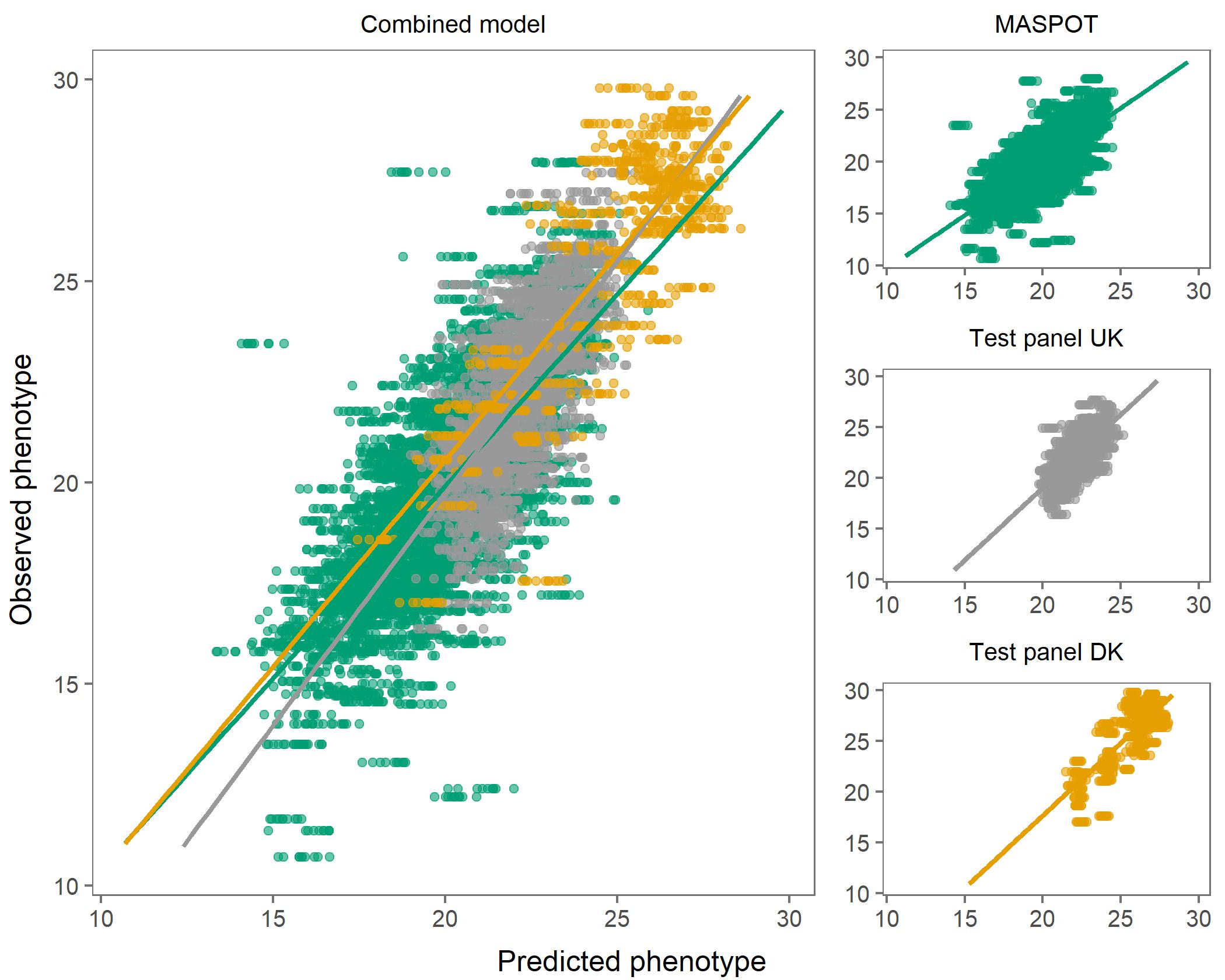
**

**Supplementary Figure S14.** **Predictions of dry matter content with 7,800 randomly selected markers.** Predictions were made using the combined model (left) or using within-population predictions (right). Green: Predictions of MASPOT population. Grey: Predictions of Test panel UK. Yellow: Predictions of Test panel DK.

**
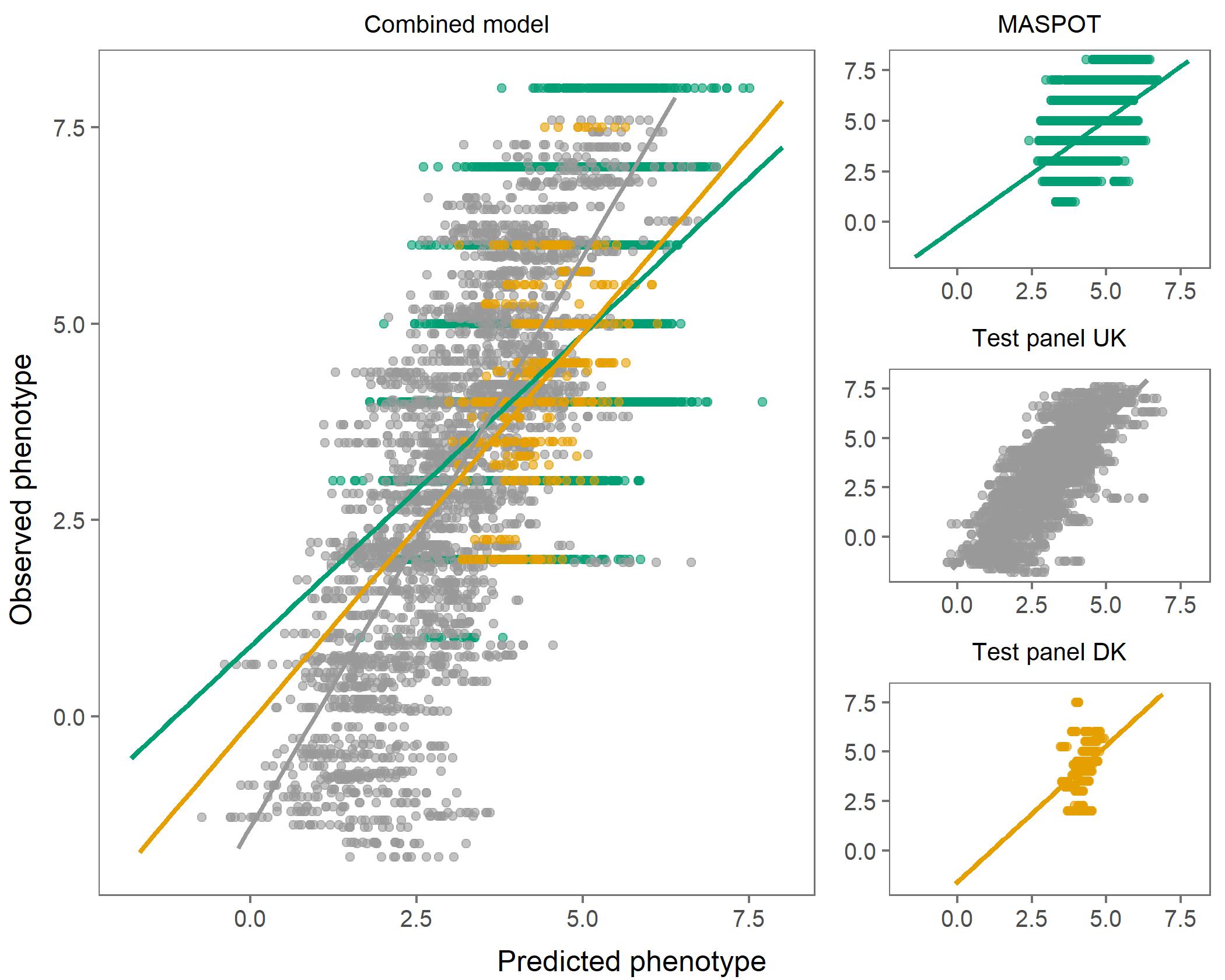
**

**Supplementary Figure S15.** **Predictions of chipping quality with 7,800 randomly selected markers.** Predictions were made using the combined model (left) or using within-population predictions (right). Green: Predictions of MASPOT population. Grey: Predictions of Test panel UK. Yellow: Predictions of Test panel DK.

**
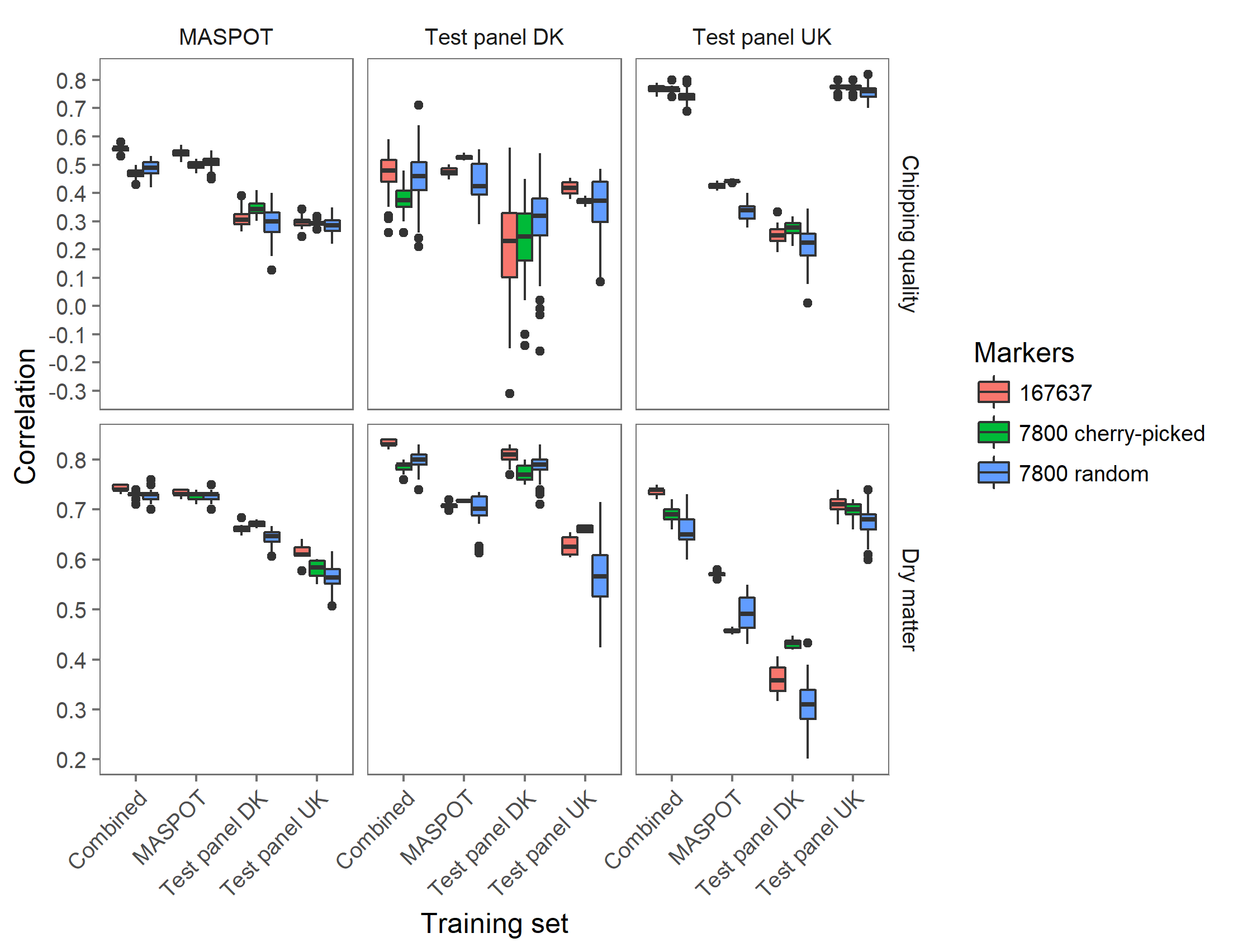
**

**Supplementary Figure S16.** **Boxplot of predictions.** Prediction correlations for the MASPOT population (left panel), Test panel DK (middle panel) and Test panel UK (right panel) for chipping quality (top) and dry matter (bottom). The training population used for the model is indicated on the x-axis. Prediction correlation for each of the 50 repeats made for each model and each population is plotted for either all 167,637 markers (red), 7,800 cherry-picked markers (green) or 7,800 random markers (blue). For the 7,800 random marker set, all 10 samplings of markers are also included, i.e. there are 10 repeats for each of the 10 marker samplings. The top and bottom of the boxes correspond to first and third quartiles, while the centerline is the median, the whiskers extend to the lowest or the highest value that is within 1.5x within the inter-quartile range, and points represent outliers.

**
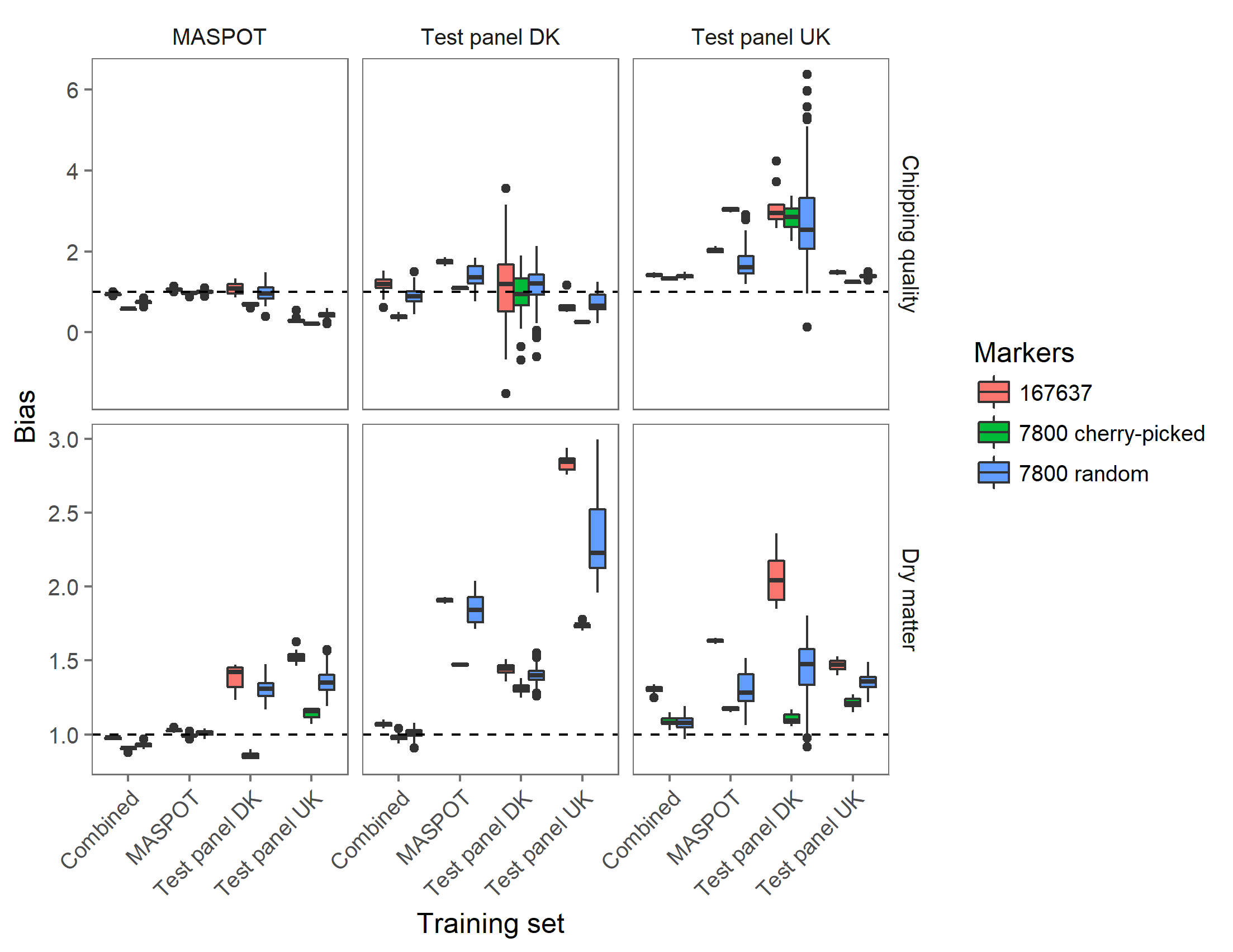
**

**Supplementary Figure S17.** **Boxplot of prediction bias.** Prediction bias for the MASPOT population (left panel), Test panel DK (middle panel) and Test panel UK (right panel) for chipping quality (top) and dry matter (bottom). The training population used for the model is indicated on the x-axis. Prediction bias for each of the 50 repeats made for each model and each population is plotted for either all 167,637 markers (red), 7,800 cherry-picked markers (green) or 7,800 random markers (blue). For the 7,800 random marker set, all 10 samplings of markers are also included, i.e. there are 10 repeats for each of the 10 marker samplings. The top and bottom of the boxes correspond to first and third quartiles, while the centerline is the median, the whiskers extend to the lowest or the highest value that is within 1.5x within the inter-quartile range, and points represent outliers. The dashed lines indicate the optimal bias of 1.

**
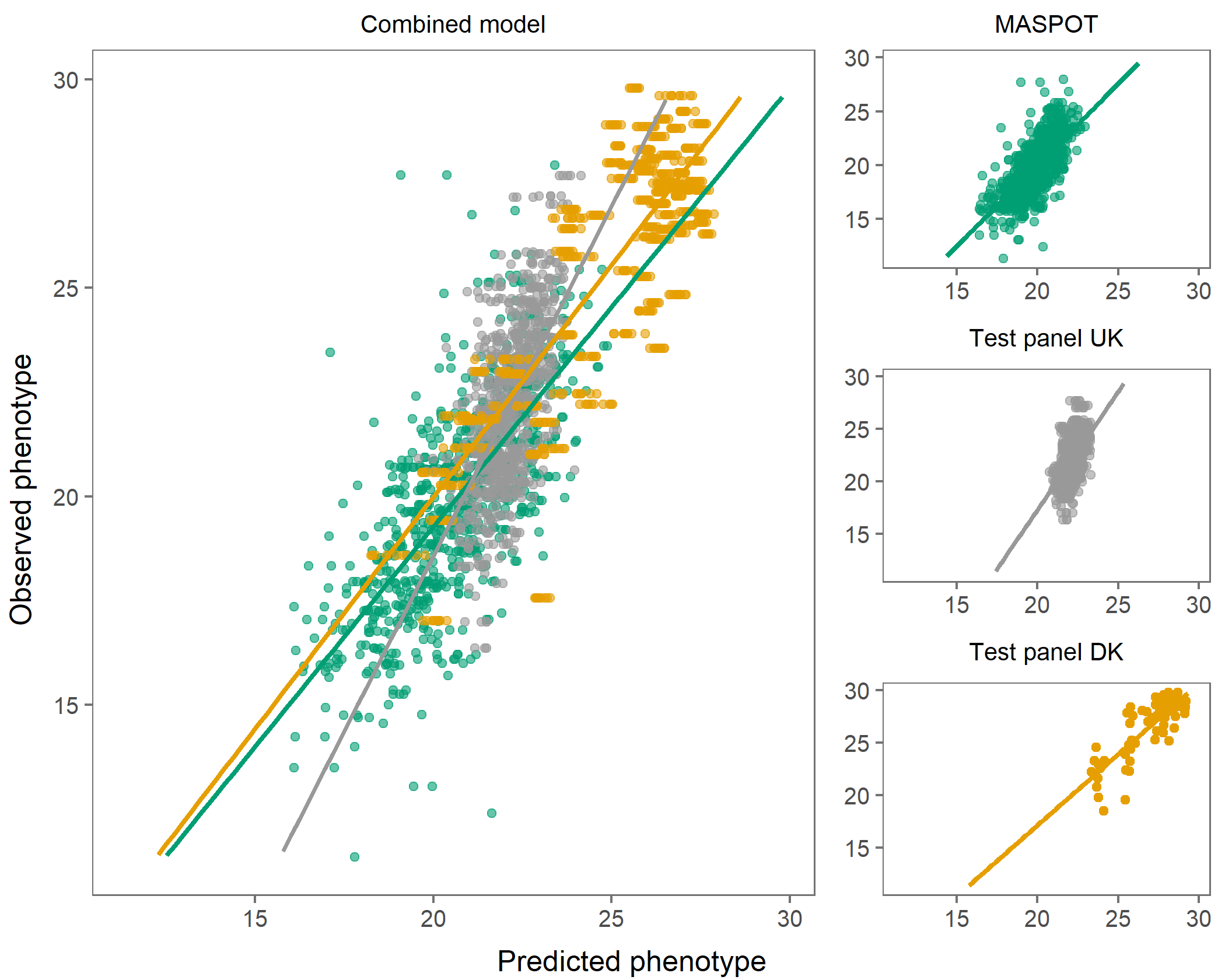
**

**Supplementary Figure S18.** **Predictions of dry matter content with subsets of 80 samples in each population.** Predictions were made using the combined model (left) or using within-population predictions (right). Green: Predictions of MASPOT population. Grey: Predictions of Test panel UK. Yellow: Predictions of Test panel DK.

**
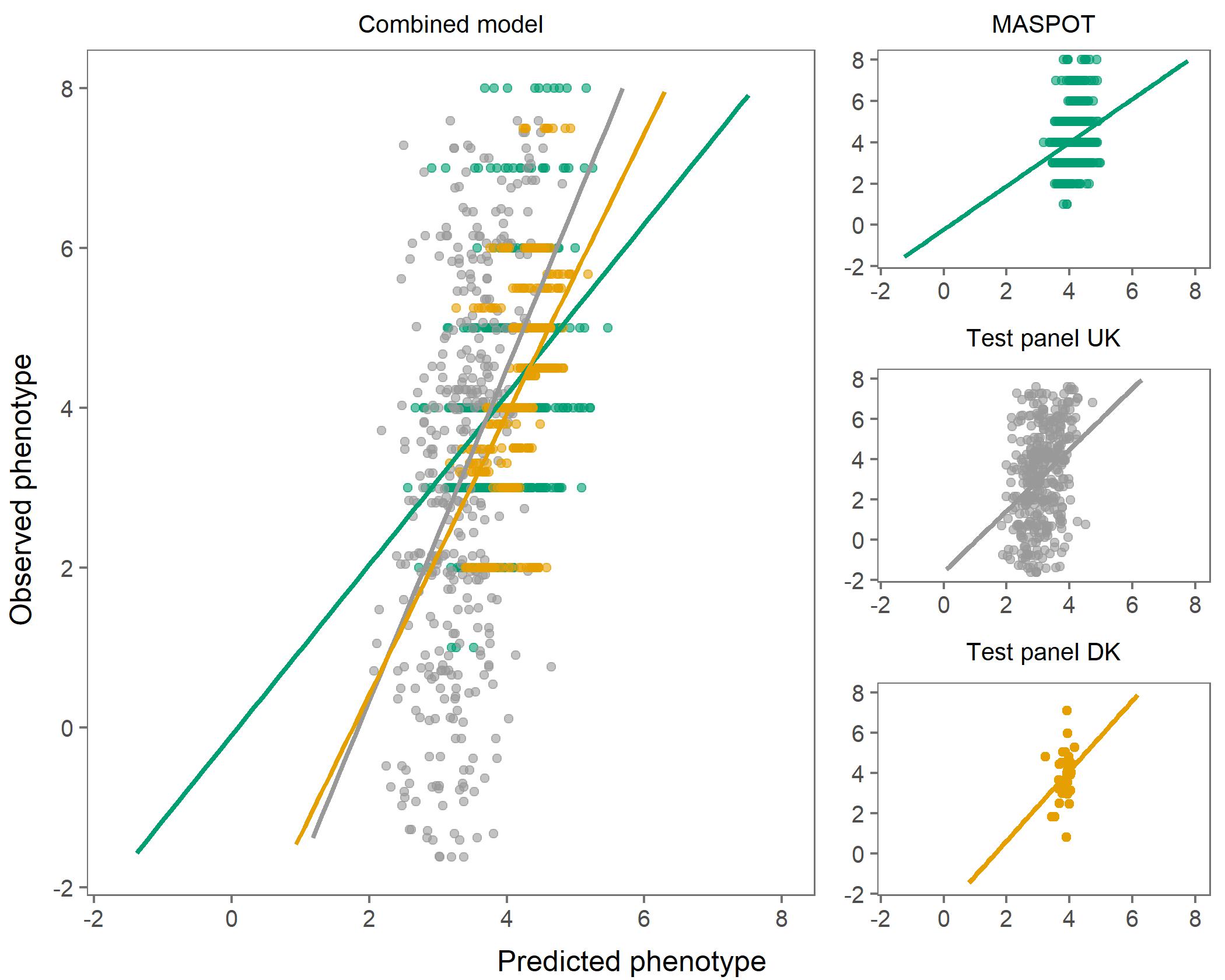
**

**Supplementary Figure S19.** **Predictions of chipping quality with subsets of 39 samples in each population.** Predictions were made using the combined model (left) or using within-population predictions (right). Green: Predictions of MASPOT population. Grey: Predictions of Test panel UK. Yellow: Predictions of Test panel DK.

**
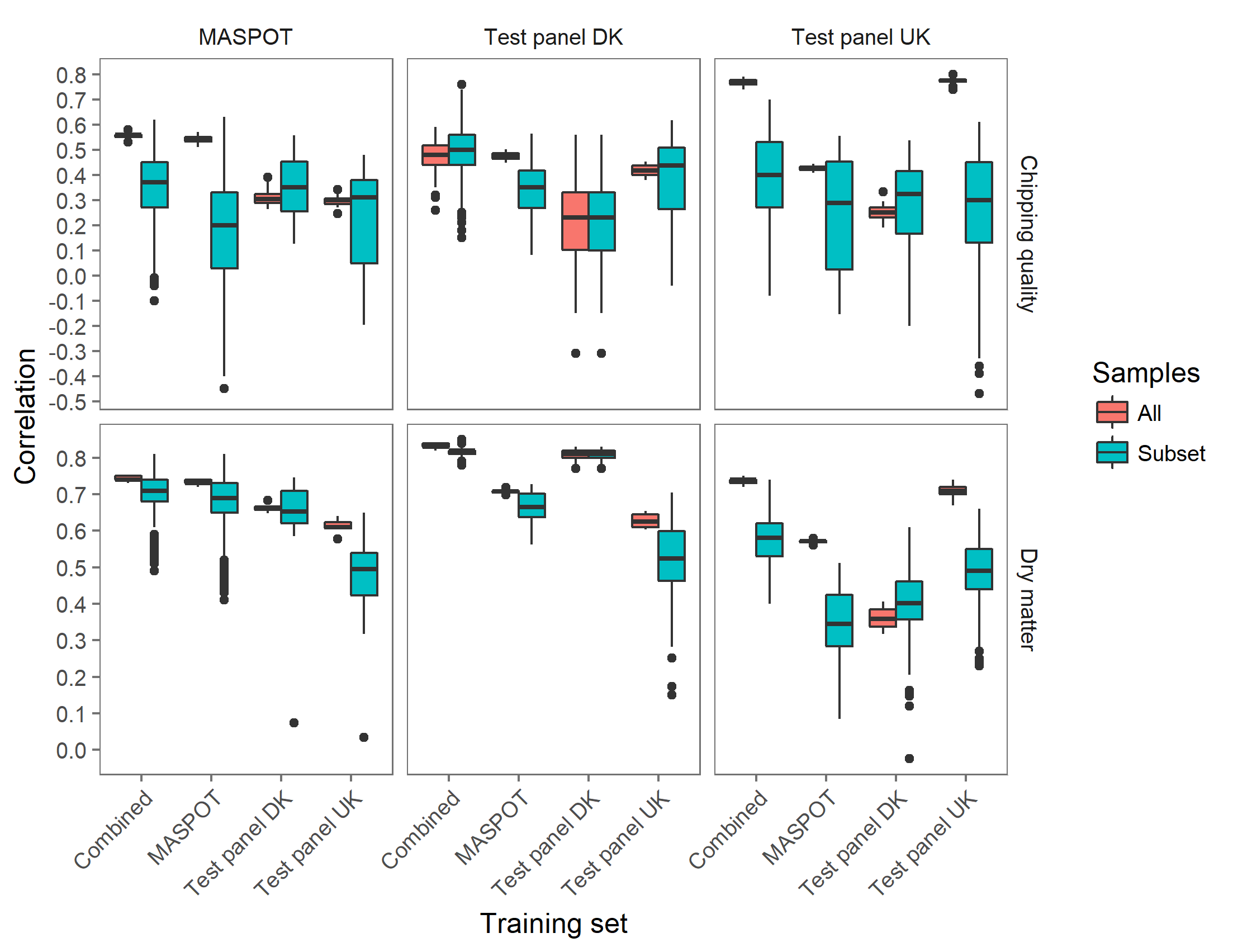
**

**Supplementary Figure S20.** **Boxplot of predictions.** Prediction correlations for the MASPOT population (left panel), Test panel DK (middle panel) and Test panel UK (right panel) for chipping quality (top) and dry matter (bottom). The training population used for the model is indicated on the x-axis. Prediction correlation for each of the 50 repeats made for each model and each population is plotted for either all samples (red) or subsets of either 80 samples (dry matter) or 39 samples (chipping quality) (green. For the subsets, all 10 samplings are also included, i.e. there are 10 repeats for each of the 10 subsets. The top and bottom of the boxes correspond to first and third quartiles, while the centerline is the median, the whiskers extend to the lowest or the highest value that is within 1.5x within the inter-quartile range, and points represent outliers.

**
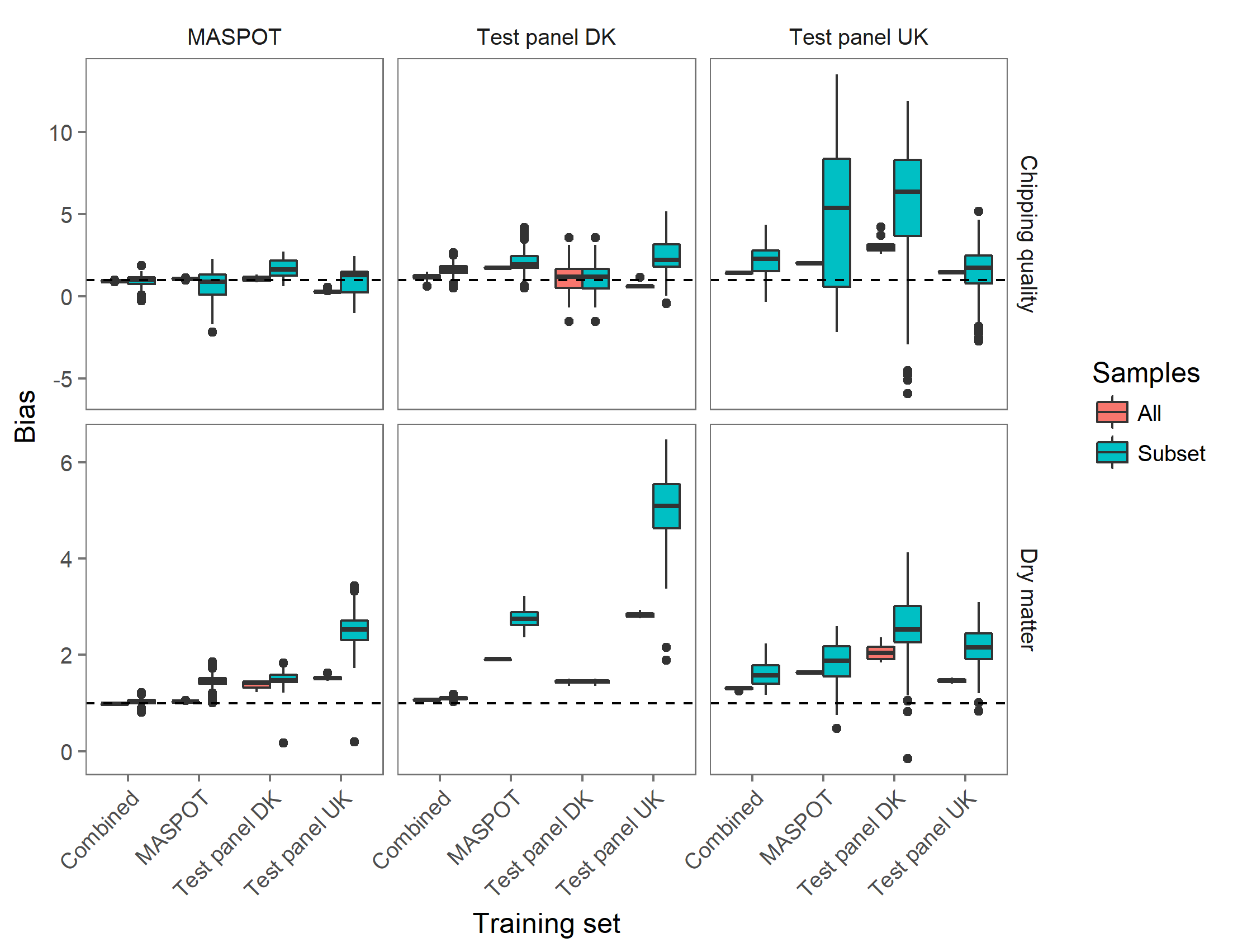
**

**Supplementary Figure S21.** **Boxplot of prediction bias.** Prediction bias for the MASPOT population (left panel), Test panel DK (middle panel) and Test panel UK (right panel) for chipping quality (top) and dry matter (bottom). The training population used for the model is indicated on the x-axis. Prediction bias for each of the 50 repeats made for each model and each population is plotted for either all samples (red) or subsets of either 80 samples (dry matter) or 39 samples (chipping quality) (green. For the subsets, all 10 samplings are also included, i.e. there are 10 repeats for each of the 10 subsets. The top and bottom of the boxes correspond to first and third quartiles, while the centerline is the median, the whiskers extend to the lowest or the highest value that is within 1.5x within the inter-quartile range, and points represent outliers. The dashed lines indicate the optimal bias of 1.
