# Supplementary material for: The Value of Expanding the Training Population to Improve Genomic Selection Models in Tetraploid Potato
Source: Front Plant Sci. 2018 Aug 6;9:1118. doi: 10.3389/fpls.2018.01118 (PMC6090097; doi:10.3389/fpls.2018.01118)
Supplement: Supplementary file 8 [file Table_1.DOCX]

Supplementary Material

The value of expanding the training population in genomic selection models for tetraploid potato

Elsa Sverrisdóttir*, Ea Høegh Riis Sundmark, Heidi Øllegaard Johnsen, Hanne Grethe Kirk, Torben Asp, Luc Janss, Glenn Bryan, and Kåre L. Nielsen

*** Correspondence:** Elsa Sverrisdóttir: esv@bio.aau.dk

# Supplementary Table S1

**Supplementary Table S1.** Prediction correlations and bias found with GBLUP with 167,637 markers within each population using a leave-one-out cross-validation system.

| Trait | MASPOT | Test panel DK | Test panel UK | Combined |
| --- | --- | --- | --- | --- |
| Chipping quality | 0.56 [1.06] | 0.33 [1.71] | 0.80 [1.41] | 0.69 [1.13] |
| Dry matter | 0.74 [1.02] | 0.81 [1.38] | 0.73 [1.40] | 0.82 [1.05] |

Bias is listed in brackets.
