# Supplementary material for: The Value of Expanding the Training Population to Improve Genomic Selection Models in Tetraploid Potato
Source: Front Plant Sci. 2018 Aug 6;9:1118. doi: 10.3389/fpls.2018.01118 (PMC6090097; doi:10.3389/fpls.2018.01118)
Supplement: Supplementary file 9 [file Table_2.DOCX]

Supplementary Material

The value of expanding the training population in genomic selection models for tetraploid potato

Elsa Sverrisdóttir*, Ea Høegh Riis Sundmark, Heidi Øllegaard Johnsen, Hanne Grethe Kirk, Torben Asp, Luc Janss, Glenn Bryan, and Kåre L. Nielsen

*** Correspondence:** Elsa Sverrisdóttir: esv@bio.aau.dk

# Supplementary Table S2

**Supplementary Table S2.** Mean prediction correlations and bias found with BayesA over 10 repeats with 167,637 markers, using the three populations separately and combined for modelling.

| Prediction set / Training set | MASPOT | Test panel DK | Test panel UK | Combined |
| --- | --- | --- | --- | --- |
| **Chipping quality** |  |  |  |  |
| MASPOT [524] | **0.56 [1.07]** ^*^ | 0.34 [1.33] | 0.32 [0.54] | 0.57 [0.96] ^***^ |
| Test panel DK [40] | 0.46 [1.64] | **0.29 [1.55]** ^**^ | 0.42 [1.08] | 0.47 [1.16] ^***^ |
| Test panel UK [290] | 0.43 [1.94] | 0.29 [3.92] | **0.79 [1.54]** ^**^ | 0.78 [1.38] ^***^ |
| **Dry matter** |  |  |  |  |
| MASPOT [755] | **0.74 [1.02]** ^*^ | 0.68 [1.39] | 0.63 [1.57] | 0.75 [0.98] ^***^ |
| Test panel DK [80] | 0.70 [1.87] | **0.82 [1.40]** ^**^ | 0.64 [2.76] | 0.83 [1.06] ^***^ |
| Test panel UK [290] | 0.58 [1.58] | 0.37 [2.16] | **0.72 [1.52]** ^**^ | 0.76 [1.31] ^***^ |

The population used for training the model is listed horizontally while the predicted population is listed vertically. Bias is listed in brackets. The number of phenotypes available in each case is indicated with brackets. Bold lettering indicates within-population predictions, where the same population was used for training and test population. ^*^8-fold cross-validation; ^**^4-fold cross-validation; ^***^12-fold cross-validation
